# Metabolomic Evaluation of Tissue-Specific Defense Responses in Tomato Plants Modulated by PGPR-Priming against *Phytophthora capsici* Infection

**DOI:** 10.3390/plants10081530

**Published:** 2021-07-26

**Authors:** Msizi I. Mhlongo, Lizelle A. Piater, Paul A. Steenkamp, Nico Labuschagne, Ian A. Dubery

**Affiliations:** 1 Research Centre for Plant Metabolomics, Department of Biochemistry, University of Johannesburg, P.O. Box 524, Auckland Park, Johannesburg 2006, South Africa; mmhlongo@uj.ac.za (M.I.M.); lpiater@uj.ac.za (L.A.P.); psteenkamp@uj.ac.za (P.A.S.); 2 Department of Plant and Soil Sciences, University of Pretoria, Private Bag X20, Hatfield, Pretoria 0028, South Africa; nico.labuschagne@up.ac.za

**Keywords:** plant growth-promoting rhizobacteria, priming, *Phytophthora capsici*, metabolic reprogramming, tomato, ultra-high performance liquid chromatography, mass spectrometry, tandem mass spectrometry

## Abstract

Plant growth-promoting rhizobacteria (PGPR) can stimulate disease suppression through the induction of an enhanced state of defense readiness. Here, untargeted ultra-high performance liquid chromatography–mass spectrometry (UHPLC–MS) and targeted ultra-high performance liquid chromatography coupled to triple-quadrupole mass spectrometry (UHPLC–QqQ-MS) were used to investigate metabolic reprogramming in tomato plant tissues in response to priming by *Pseudomonas fluorescens* N04 and *Paenibacillus alvei* T22 against *Phytophthora capsici*. Roots were treated with the two PGPR strains prior to stem inoculation with *Ph. capsici.* Metabolites were methanol-extracted from roots, stems and leaves at two–eight days post-inoculation. Targeted analysis by UHPLC–QqQ-MS allowed quantification of aromatic amino acids and phytohormones. For untargeted analysis, UHPLC–MS data were chemometrically processed to determine signatory biomarkers related to priming against *Ph. capsici*. The aromatic amino acid content was differentially reprogrammed in *Ps. fluorescens* and *Pa. alvei* primed plants responding to *Ph. capsici*. Furthermore, abscisic acid and methyl salicylic acid were found to be major signaling molecules in the tripartite interaction. LC–MS metabolomics analysis showed time-dependent metabolic changes in the primed-unchallenged vs. primed-challenged tissues. The annotated metabolites included phenylpropanoids, benzoic acids, glycoalkaloids, flavonoids, amino acids, organic acids, as well as oxygenated fatty acids. Tissue-specific reprogramming across diverse metabolic networks in roots, stems and leaves was also observed, which demonstrated that PGPR priming resulted in modulation of the defense response to *Ph. capsici* infection.

## 1. Introduction

In addition to facilitating the absorption of water and minerals, roots secrete organic exudates into the rhizosphere [1,2]. The composition of root exudates plays an important role in microbial population attraction and selection of the microbial community colonizing the rhizosphere [1,3,4]. The latter comprises of neutral, pathogenic and beneficial microbes. Among these are the plant growth-promoting rhizobacteria (PGPR) that have beneficial effects on plant growth, development and production via direct (by synthesizing phytohormones, or facilitating the uptake of certain nutrients from the environment) or indirect (by producing antagonistic substances or inducing resistance to pathogens) mechanisms [5,6,7]. In addition, root colonization by PGPR may initiate a primed state of defense readiness against impending infection [2], potentially resulting in induced systemic resistance (ISR) effective against a broad spectrum of pathogens [5,8,9]. Compared to costly defense responses that may stunt plant growth and lower agricultural yields, the triggering of ISR following defense priming may have less of a detrimental effect [7,10]. The utilization of beneficial microbes is thus considered a “green”/non-agrochemical approach to augment plant immunity and to control diseases and is referred to as “bio-priming” with PGPR.

The interaction of plant roots with microorganisms within the rhizosphere involves the perception of “non-self” signature molecules (microbe-associated molecular pattern molecules or MAMPs), which activates MAMP-triggered immunity (MTI) [11,12,13]. As such, the ability of potential pathogens to cause disease is closely related to the host’s ability to recognize “non-self” metabolites and respond to these. Numerous studies have investigated the induction of ISR by PGPR strains, and mechanisms such as the recognition of MAMPs and phytohormones secreted by PGPR have been identified as priming events [2,14]. However, plant immunity can also be triggered by other microbial metabolites [8,15,16,17,18,19,20,21,22,23]. Thus, when interacting with PGPR, plants employ similar mechanisms for the perception of pathogens; however, since there are no further signals of infection, signs of cell damage or presence of microbial DNA or RNA, the initial response is deactivated, but the memory of the event retained [2,24,25,26].

Phytohormones (either endogenously synthesized or PGPR-derived) may accumulate in varying amounts upon microbe detection or infection and lead to activation of defense genes, reprogramming of the transcriptome and production of phytoalexins. To minimize fitness costs of activating unnecessary defense genes and to specify the defense response, phytohormones are produced that are specific to the stress detected [27,28,29]. In this regard, salicylic acid (SA)-induced resistance is more effective against biotrophic pathogens while jasmonic acid (JA)- and/or ethylene (ET)-induced resistance is operative against necrotrophic and herbivore attack [30]. Accordingly, different plant species can employ diverse signal transduction pathways to specify an immune response and, with the said hormones interacting either antagonistically or synergistically, modulate the reprogrammed defense output.

The oomycete *Phytophthora capsici* is a devastating hemibiotrophic pathogen, spread via irrigation and other agricultural practices. It has a broad host range [31,32,33], and produces several symptoms, such as foliar blighting, damping-off, wilting, and root-, stem- and fruit rot [31,34]. The agricultural industry relies on chemical fungicides in combination with crop rotation and soil management to help reduce the occurrence and severity of *Phytophthora* diseases [35,36]. However, the host range of *Ph. capsici* and thick zoospores limit control measures. In addition, because of the ability of *Ph. capsici* to quickly overcome resistant crop varieties and fungicides due to frequent sexual reproduction, a better understanding of host defense responses to infection would allow for more effective deployment of resistant varieties and breeding for durable resistance [34,37]. In addition, due to the negative impact of fungicides on the environment, PGPR-based treatment could be a potential solution.

Metabolite profiling of primed/challenged and naïve plants can provide more insights into PGPR priming and here metabolomics tools and approaches have contributed to novel insights into the priming phenomenon [2,38,39,40]. Previously, we investigated the metabolic reprogramming that resulted from PGPR treatment of tomato roots, where the metabolic alterations in stems and leaves pointed to an altered or pre-conditioned state that may have potentially rendered the plants primed for enhanced defense responses [41]. Next, inducible defense responses of naïve (non-primed) tomato plants toward *Ph. capsici* infection defined the chemical space of major classes of reprogrammed metabolites as comprising of phenylpropanoids, benzoic acids, glycoalkaloids, flavonoids, amino acids, organic acids and fatty acids [42]. In the present study, untargeted and targeted metabolomics approaches based on ultra-high performance liquid chromatography coupled to mass spectrometric detection (UHPLC-MS) were combined to study the inducible metabolic responses of tomato plants (pre-primed with *Pseudomonas fluorescens* N04 and *Paenibacillus alvei* T22) and subsequently challenged with *Ph. capsici*. Since the metabolome is strongly associated with the phenotype (reflecting the genotype-directed transcriptome and proteome, changes in enzyme activities and metabolic fluxes), qualitative metabolomic analysis would thus informatively reflect differential and functional features of the primed vs. naïve defense responses previously reported on [42]. Such studies would point out metabolic pathways involved in PGPR priming in tomato against *Ph. capsici*, thus contributing to the efforts to unravel the biochemical and molecular mechanism thereof.

## 2. Results

### 2.1. Symptom Development

**Figure 1:**
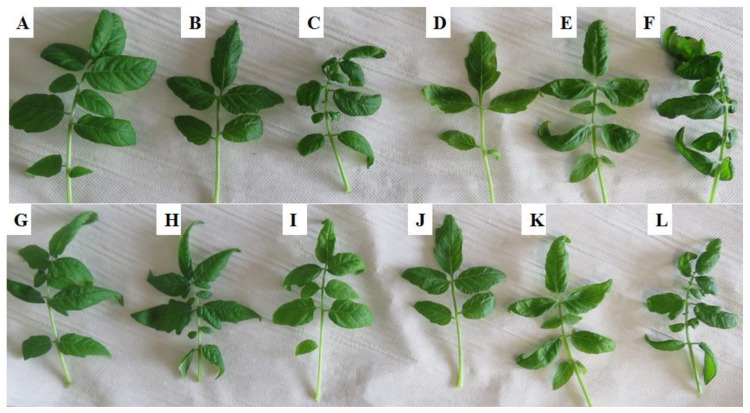
Symptom development in PGPR-primed tomato leaves harvested at different time points following infection with *Phytophthora capsici* zoospores. (**A**,**B**) *Pseudomonas fluorescens* N04 primed-unchallenged, days 2 and 8; (**C**–**F**) *Ps. fluorescens* primed-challenged—2, 4, 6 and 8 d.p.i.; (**G**,**H**) *Paenibacillus alvei* T22 primed-unchallenged, days 2 and 8; (**I**–**L**) *Pa. alvei* primed-challenged—2, 4, 6 and 8 d.p.i.

*Ph. capsici* is a hemibiotrophic microorganism involving a biotic trophic phase while establishing infection and a necrotrophic phase, which kills the host plant [43,44,45]. Two days post-PGPR inoculation, the plants were infected with *Ph. capsici* and progression of symptoms were monitored. Previous studies have shown that symptom progression involves ascending wilting, stem rot and canopy wilting [32,42]. In the companion paper [42] (conducted under the same conditions, but executed as a separate study with no overlapping data), symptoms were observed four to eight days post infection, with day 8 showing completely diseased plants. Here, PGPR-primed plants showed either reduced or no infection upon inoculation with *Ph. capsici* during the period 2–8 d.p.i. (Figure 1). In the case of *Ps. fluorescens* primed-*Ph. capsici* challenged plants, signs of wilting on the edges of leaves were observed at 8 d.p.i. (Figure 1F), whereas *Pa. alvei* primed and *Ph. capsici* challenged plants displayed no sign of wilting on 8 d.p.i. (Figure 1L). No yellowing or wilting was observed on the primed-unchallenged control plants. Thus, the results demonstrate that PGPR-inoculation leads to biochemical changes within the tomato plants, phenotypically observable as induced resistance against *Ph. capsici*.

### 2.2. Aromatic Amino Acid Quantification

**Figure 2:**
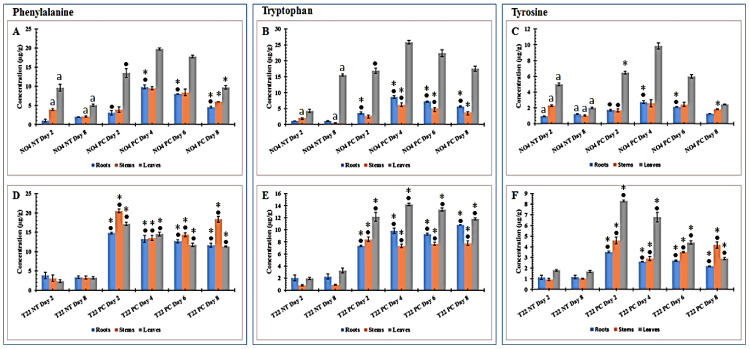
Changes in aromatic amino acids in PGPR primed tomato plant tissues harvested at different time points following *Phytophthora capsici* inoculation. (**A**–**C**) *Pseudomonas fluorescens* N04 primed-unchallenged (NT) vs. *Ps. fluorescens* primed-challenged (PC), (**D**–**F**) *Paenibacillus alvei* T22-primed-unchallenged vs. *Pa. alvei* primed-challenged. All concentrations are expressed as µg/g fresh weight (FW). Non-treated (NT) controls at the start (day 2) and end (day 8) of the time series were included. Comparing the PGPR-primed-unchallenged plants and the primed-infected plants, a dot (•), asterisk (*) or letter (a) indicates the statistical significance (ANOVA followed by Tukey post-hoc test at a *p*-value < 0.05) (Table S5): • indicates a comparison between NT day 2 and the various primed-challenged plants on days 2–8 and * indicates a comparison between NT day 8 and the various primed-challenged plants on days 2–8. The letter “a” indicates a statistically significant difference between the NT controls on days 2 and 8. The results show a time-dependent differential reprogramming of aromatic amino acid levels by *Ph. capsici* in the various tissues.

Aromatic amino acids were quantified using an UHPLC–triple quadrupole–MS multiple reaction monitoring (MRM) method applied to extracts from tomato plant tissues (root, stem and leaf) of PGPR-primed-unchallenged (NT) vs. PGPR-primed-challenged (PC) plants. The phenylalanine (Phe) contents were found to be lower in both PGPR-primed-unchallenged (*Ps. fluorescens* NT day 2 and 8 and *Pa. alvei* NT day 2 and 8) samples for all tissues. When the primed plants were challenged by *Ph. capsici*, Phe accumulated, albeit variably, over time (Figure 2A,D) compared to the unchallenged counterparts. Here, the Phe content reached a maximum on day 4 post-challenge in *Ps. fluorescens* primed plants, while for the comparable *Pa. alvei* primed plants, Phe levels reached a maximum on day 2 post-challenge, followed by a slight decrease until day 8, excluding the stem samples. In the defense response of naïve plants responding to *Ph. capsici* infection [42], the Phe content showed a gradual increase over the same time period investigated in this study. The rapid accumulation (especially in the case of *Pa. alvei* primed-challenged plants) indicates that pre-treatment with the PGPR pre-conditioned the synthesis of Phe for enhanced and quick accumulation of upon *Ph. capsici* infection.

A similar trend was observed for tryptophan (Trp) in extracts from *Ps. fluorescens* primed-challenged plants, where a maximum accumulation was reached on day 4, followed by a slight decrease thereafter (Figure 2B). Moreover, for the NT controls on day 8 there was a notable difference from day 2. In *Pa. alvei* primed-challenged plants, the Trp content reached a maximum accumulation on day 2 for stem tissue, day 4 for leaves and day 8 for roots (Figure 2E). Thereafter, the Trp content did not show any decrease but rather remained approximately the same over time following challenge. The metabolic defense response of naïve tomato plant tissues (leaf, stem and root) to *Ph. capsici* infection showed a continuous increase in Trp content reaching a maximum on day 4 and followed by a slight decrease thereafter in the various tissues [42]. When compared to primed-challenged plants it is evident that PGPR inoculation (especially *Pa. alvei* T22) pre-conditioned the Trp biosynthesis pathway for quicker accumulation when challenged by a secondary stimulus.

Following challenge of the *Ps. fluorescens* primed plants, the tyrosine (Tyr) content was only found to be modified in leaf tissue, where maximum accumulation was reached on day 4 followed by a steady decrease thereafter. Moreover, NT day 2 was higher than NT day 8, which indicates that there is a decrease over time regardless of the challenge. In root and stem tissues, the Tyr content was not affected by *Ph. capsici* infection (Figure 2C). In the counterpart *Pa. alvei* primed plants, the Tyr content followed that of Phe (Figure 2D) and Trp (Figure 2E) subsequent to challenge, reaching maximum accumulation on day 2 (T22 PC day 2) and a steady decrease over time thereafter (Figure 2F). The metabolic response of *Ps. fluorescens* primed-challenged plants (Figure 2C) was found to be similar to the metabolic response of naïve plants infected with *Ph. capsici* [42]. This indicates that *Ps. fluorescens* inoculation does not affect the Tyr biosynthesis pathway. However, *Pa. alvei* primed-challenged plants showed a rapid accumulation reaching a maximum on day 2 (Figure 2F), indicating that *Pa. alvei* inoculation sensitized or conditioned the Tyr biosynthesis pathway for quicker accumulation.

One-way ANOVA comparing mean values of quantified aromatic amino acids in PGPR-primed-unchallenged vs. PGPR-primed-challenged tomato plant tissues is presented in Table S1.

Previously we reported on the metabolic perturbations by PGPR inoculation and it was observed that *Pa. alvei* and *Ps. fluorescens* had a differential effect on the content of aromatic amino acids, characterized by an increase or decrease [41]. Here, pre-treatment with *Pa. alvei* followed by infection was characterized by the strong and rapid accumulation of the aromatic amino acids, whereas *Ps. fluorescens* pre-treatment followed by infection had little effect on the actual contents of Phe, Trp and Tyr. This can be interpreted that the induction of ISR by *Pa. alvei* involves the sensitization of the aromatic amino acid biosynthetic pathways. Moreover, the observed differential perturbation by the two strains further suggests different mechanisms of action with regards to induction of ISR.

### 2.3. Abscisic Acid (ABA) and Methyl Salicylic Acid (MeSA) Quantification

**Figure 3:**
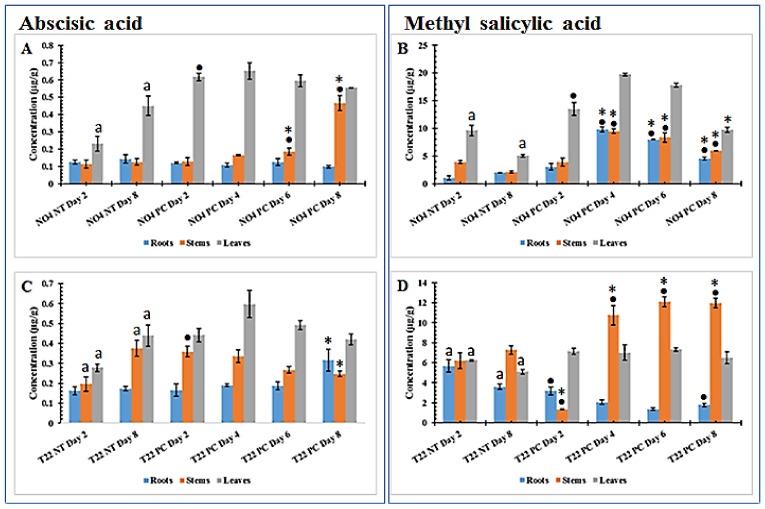
Changes in abscisic acid (**A**,**C**) and methyl salicylic acid (**B**,**D**) in PGPR-primed tomato plant tissues harvested at different time points following *Phytophthora capsici* inoculation. (**A**,**B**) *Pseudomonas fluorescens* N04 primed-unchallenged (NT) vs. primed-challenged (PC), (**C**,**D**) *Paenibacillus alvei* T22-primed-unchallenged vs. primed-challenged. All concentrations are expressed as µg/g fresh weight (FW). An asterisk (*) or a dot (•) or letter (a) indicates the statistical significance (ANOVA followed by Tukey post-hoc test) with a *p*-value < 0.05 (Table S5), comparing the PGPR-primed-unchallenged plants and the primed-infected plants. • indicates a comparison between NT day 2 and the primed-challenged plants on days 2–8 and * indicates a comparison between NT day 8 and the primed-challenged plants on days 2–8. The letter “a” indicates a statistically significant difference between the NT controls on days 2 and 8. One-way ANOVA comparing mean values of quantified phytohormones in PGPR-primed-unchallenged vs. PGPR-primed-challenged tomato plant tissues is presented in Table S1.

Changes in phytohormones in plants primed with *Ps. fluorescens* (Figure 3A,B) and *Pa. alvei* (Figure 3C,D) followed by challenge with were quantified as described [42]. Technical aspects of the MRM assays are presented in Tables S2–S4. ABA and MeSA were two phytohormones or signaling molecules found to be upregulated in the primed plants in response to *Ph. capsici* and gave reproducible results above the limit of quantification.

The ABA content was found to not be affected in root—and stem tissue of the challenged *Ps. fluorescens* primed samples (no clear differences were observed between primed-unchallenged and primed-challenged), with only a significant increase in accumulation observed on days 6 and 8 (N04 PC treated plants) in stems (Figure 3A) when compared to the primed-only state. In leaf tissue, a statistical difference was only observed when comparing N04 NT day 2 and N04 PC day 2. For *Pa. alvei* T22-primed-unchallenged (T22 NT day 2 and T22 NT day 8) plants, a significant difference was observed in stems and leaves when compared with each other (Figure 3C). As such, only day 2 and day 8 controls were compared with the treatment of the same time points (i.e., T22 NT day 2 vs. T22 PC day 2 and T22 NT day 8 vs. T22 PC day 8). Only stem tissue comparison shows significant accumulation of ABA between N04 NT day 2 vs. N04 PC day 8. However, in leaf tissue of plants primed with both PGPR strains, no significant accumulation was observed for the specific time point comparison.

The MeSA content increased with time subsequent to challenge in *Ps. fluorescens* primed plants, reaching a maximum on day 4 in roots and stem tissues and a slight decrease until day 8, but higher than in the unchallenged plants (Figure 3B). However, for leaf tissue the two controls had a significant difference between them and as such they were compared to the corresponding time points. Significant differences were observed between these time points. In *Pa. alvei* primed plants both roots and leaf tissue controls had a significant difference and they compared to their counterpart time points (Figure 3D). In roots, both time points comparison (T22 NT day 2 vs. T22 PC day 2 and T22 NT day 8 vs. T22 PC day 8) showed significantly lower levels of MeSA in *Pa. alvei* primed challenged plants when compared to the corresponding primed-unchallenged plants. In contrast, in leaf tissue there was no significant difference between the compared time points (T22 NT day 2 vs. T22 PC day 2 and T22 NT day 8 vs. T22 PC day 8) (Figure 3D). In stem tissue, MeSA content showed a significant accumulation over time from day 4 to day 8 in *Pa. alvei* primed-challenged plants when compared to the corresponding primed-unchallenged plants. This differential regulation of ABA and MeSA content in PGPR-primed plants indicates priming to be highly dependent on the priming agent, which may also be true for the response to secondary stresses.

In our previous study of naïve plants infected with *Ph. capsici*, only MeSA and 1-aminocyclopropane-1-carboxylate (ACC) were successfully quantified. MeSA was found to be differentially regulated, whereas ACC showed a continuous increase over time [43]. Here, ABA and MeSA were the only two phytohormones successfully quantified. ABA was slightly affected in both PGPR-primed-unchallenged and PGPR-primed-challenged plants. However, MeSA was increased in *Ps. fluorescens* primed-challenged plants when compared to unchallenged plants, thus indicating that pre-treatment with *Ps. fluorescens* priming/sensitizing may involve salicylic acid biosynthesis and methylation. In *Pa. alvei* primed-challenged compared to the corresponding primed-unchallenged plants, MeSA was found to increase in the stem tissue, while in root tissue it decreased and in leaf tissue no significant difference was observed (Figure 3D). The differential perturbation of phytohormones observed with the respective PGPR treatments suggests that the defense responses are fine-tuned based on the perceived stimuli.

### 2.4. Metabolomic Profiling of PGPR-Primed Plants Responding to Phytophthora capsici

**Figure 4:**
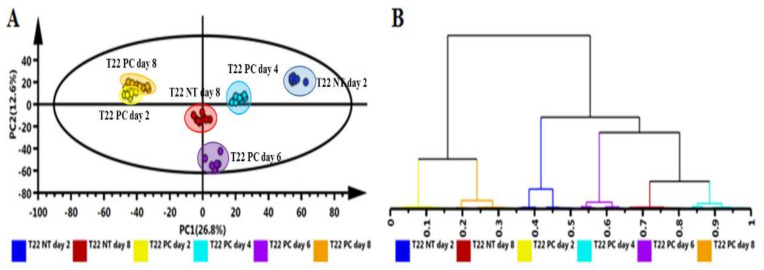
Unsupervised statistical modeling of leaf data comparing plants primed by *Paenibacillus alvei* T22 and primed and challenged with *Phytophthora capsici.* (**A**) A principal component analysis (PCA) scores scatter plot of all the samples, including the QC samples, colored according to time points: T22 NT, day 2 (blue) and day 8 (brown) and T22 inoculated and PC treated, days 2 (yellow), 4 (teal), 6 (purple) and 8 (orange). The PCA model presented here was a 7-component model, with R^2^ of 0.739 and Q^2^ of 0.664. (**B**) The hierarchical cluster analysis (HiCA) dendrogram corresponding to (**A**). Unsupervised statistical analysis is used to generate subgrouping of samples based on similar observations in (**A**) while the HiCA dendrogram shows the hierarchical relationship between samples (**B**). Data were acquired in (–) electrospray ionization mode.

Metabolomics has demonstrated that plant defense responses are associated with reprogramming of various primary and secondary metabolites. Extracts from plant samples contain thousands of chemically and structurally diverse molecules that need to be chromatographically separated prior to ESI–MS analysis and detection. Base peak intensity (BPI) chromatograms of the primed-unchallenged and primed-challenged plant extracts showed chromatographically distinct metabolite profiles with some variation in peak intensities, accumulation of new peaks and disappearance of others, e.g., Supplementary Figures S1–S3 (leaves, stems and roots, respectively) of *Pa. alvei* primed-unchallenged vs. the corresponding primed-challenged plants. These differences are also reflected in the unsupervised statistical analysis indicating subgrouping of samples in Figure 4A and the hierarchical relationship between samples in Figure 4B. This is an indication that *Pa. alvei* primed-unchallenged vs. *Pa. alvei* primed-challenged plants differed in aspects of cellular metabolism, resulting in altered, time-dependent metabolic changes. The same was observed for *Ps. fluorescens* primed-unchallenged vs. the corresponding primed-challenged plants (BPI chromatograms not shown).

LC–MS metabolite profiling data consist of thousands of multidimensional features and are inherently very complex. To reduce this complexity, chemometric analyses were employed. These included both unsupervised, with principal component analysis (PCA) and hierarchical cluster analysis (HiCA), and supervised modeling using group memberships as the categorical Y-variable in orthogonal projection to latent structures discriminant analysis (OPLS-DA). For exploration of the multidimensional data, PCA- and HiCA models were used. These aim at identifying trends and patterns within the data set, thus pinpointing relationships between and within samples. PCA models revealed a time- and treatment-dependent clustering (Figure 4A and Figures S4A–S8A). Furthermore, the quality control (QC) pooled samples were found to cluster closely to each other in the middle of the PCA score plot which reflect instrument reliability, stability and data reproducibility.

PCA trends were further examined by HiCA using Ward’s linkage method, considering distance clusters between- and within-samples (Figure 4B and Figures S4B–S8B). These were generated to evaluate subgroupings present within the data set. The computed HiCA models depicted two major distinct groupings corresponding to various time points (d.p.i.), and treatment subgrouping was observed. By itself, both PCA and HiCA assisted in evaluating the overall structure of the data sets, revealing underlying patterns, trends and subgrouping related to treatment and time of harvesting. These observations highlight aspects of the biochemical phenomena (altered metabolomic states) attributed to PGPR priming against *Ph. capsici*.

**Figure 5:**
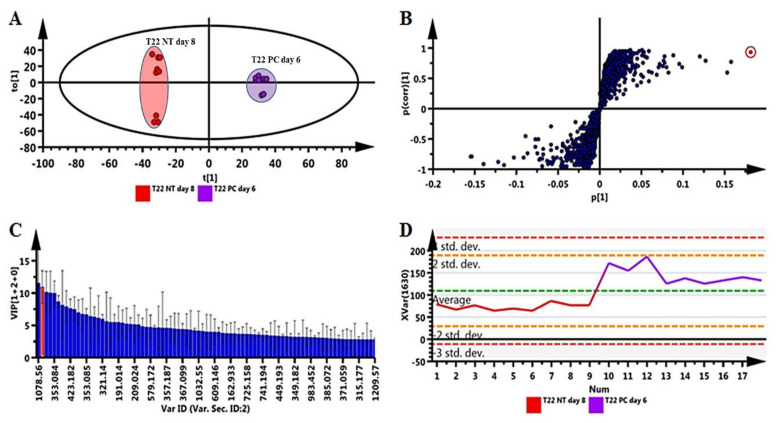
Pairwise comparison via orthogonal projection to latent structures discriminant analysis (OPLS-DA) modeling and variable selection from leaf data comparing primed (*Paenibacillus alvei* T22) and primed-challenged (*Pa. alvei*/*Phytophthora capsici*-infected) plants. (**A**) A typical score plot separating primed (T22 NT day 8) plants vs. primed-challenged (T22 PC day 6) plants (1 + 1 + 0 components, R^2^X = 0.581, Q^2^ = 0.984, CV-ANOVA *p*-value = 3.1 × 10^−^^11^). (**B**) A loadings S-plot for the same model in (**A**); only variables with a correlation ((*p*(*corr*)) ≥ |0.6| and covariance (*p1*) ≥ |0.05| were chosen as discriminating features and identified using the *m*/*z* to generate an elemental composition. (**C**) Variable importance for the projection (VIP) plot for the same model, pointing mathematically to the importance of each variable in contributing to group separation in the OPLS-DA model. (**D**) A typical variable trend plot (of the selected variable in VIP and S-plots), displaying the changes of the selected variables across the samples (NT day 8 vs. PC day 6). This shows that the selected features significantly discriminate the primed-challenged from primed-unchallenged samples. Data were acquired in ESI(–) mode.

Pairwise comparative OPLS-DA models were computed for mass spectrometric (MS) data from leaves (comparing *Pa. alvei* T22 PC day 6 to the most suitable NT control, day 8, so as to relate to the naïve (*Ph. capsici* infection only) plant study [42]) of the ESI-negative data for plants primed with *Pa. alvei* (Figure 5). The optimal number of latent variables for each model was evaluated using cross-validation (CV) with the outfit routine. The quality of the models was evaluated with routine seven-fold CV based on residuals with R^2^X(cum) (cumulative modeled variation in X), R^2^Y(cum) (cumulative modeled variation in Y) and Q^2^cum (model predictive power based on the CV). The models were tested for overfitting with CV-ANOVA (*p*-value < 0.05) based on the CV predictive residuals [46]. The computed models are presented in Figure 5 and further confirm the treatment- and time-dependent grouping shown in the PCA and HiCA plots (Figure 4) (i.e., primed vs. primed-challenged) (Figure 5A).

The OPLS-DA loading S-plot (Figure 5B) and the variable importance in projection (VIP) plot (Figure 5C) were used to demarcate the variables that were responsible for the sample grouping between the prime-unchallenged (*Pa. alvei*—NT day 8) vs. primed-challenged (*Pa. alvei*—PC day 6). Only variables with a VIP score of one or higher were rated as important [46,47,48]. Lastly, a variable trend plot (Figure 5D) of a selected variable (highlighted in red in the S-plot and VIP plot) was computed to evaluate the change of the selected feature across the samples (*Pa. alvei*—PC day 6 vs. *Pa. alvei*—NT day 8). The highlighted variables are significant elements for the biochemical interpretation underlying sample grouping. These variables were annotated to Metabolomics Standard Initiative (MSI) level 2 [49] and are presented in Table S6. Similar OPLS-DA models of analyzed data from extracts obtained from roots and stems are also presented as Figures S9–S13.

### 2.5. Time-Course of Comparative Metabolite Reprogramming in Primed-Unchallenged vs. Primed-Challenged Plants Inoculated with Phytophthora capsici

**Figure 6:**
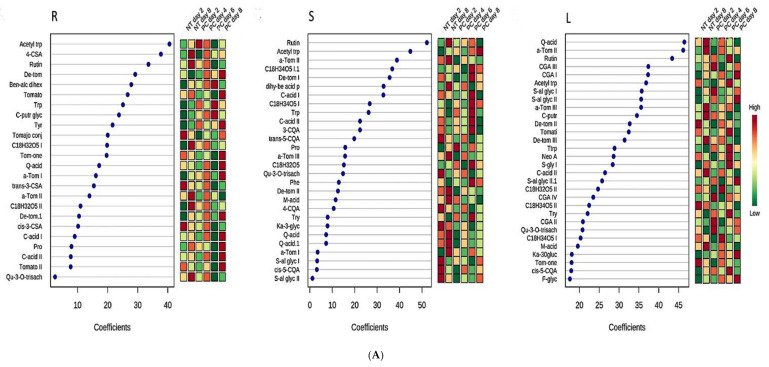

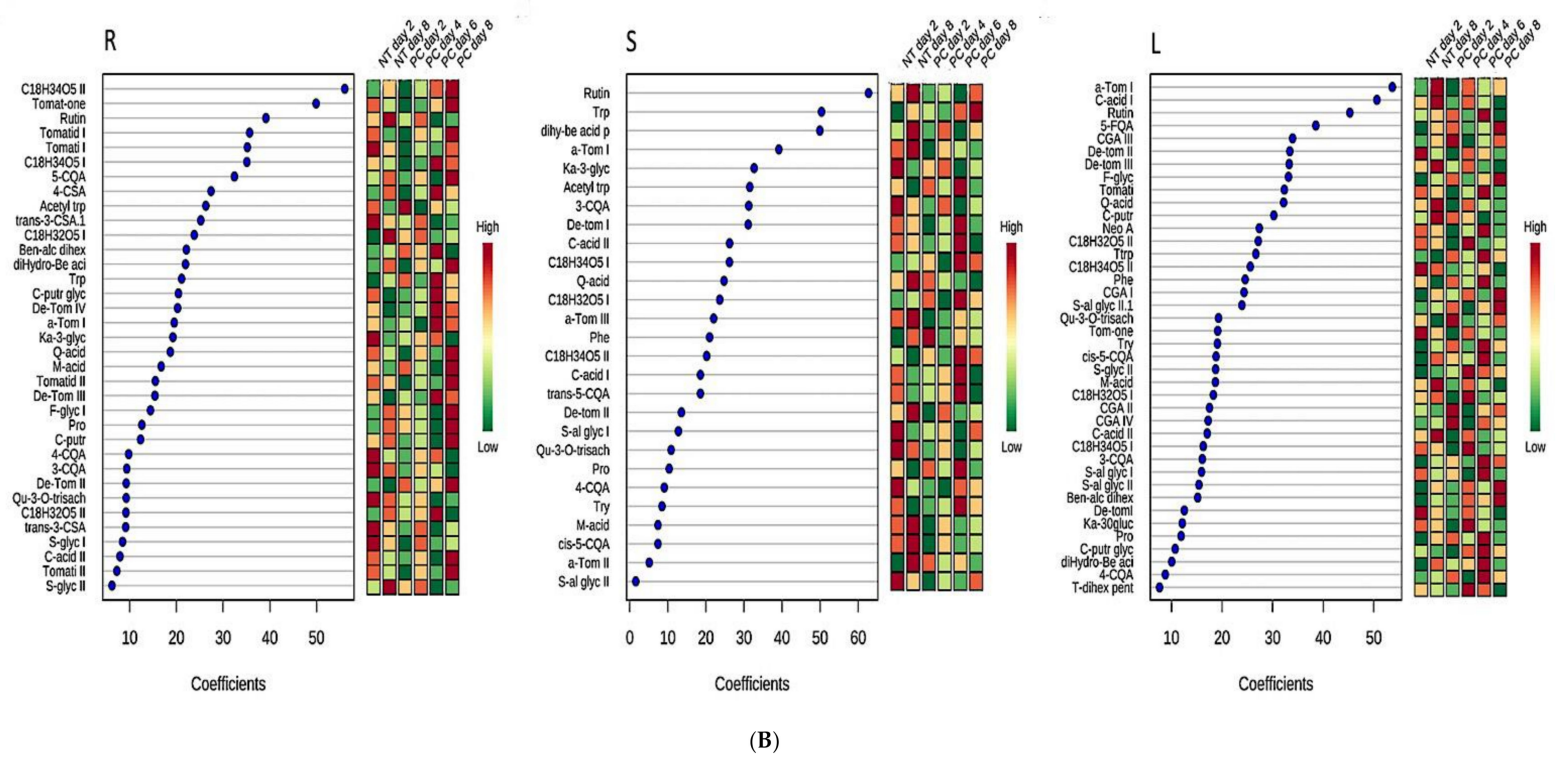
(**A**) Coefficient scores derived from the partial least squares discriminant analysis (PLS-DA) displaying discriminant features. Shown are annotated metabolites in primed-unchallenged (*Paenibacillus alvei* T22) and primed-challenged (*Phytophthora capsici*, PC) plant tissues for root (R), stem (S) and leaf (L) tissues. Selected metabolites (coefficient scores ≥ 2) from primed-unchallenged plants (NT days 2 and 8) were compared to those in primed-challenged plants at the given time point (PC—2, 4, 6, 8 d.p.i.). The abbreviated names of the annotated metabolites are listed in Table S6. (**B**) Coefficient scores derived from the partial least squares discriminant analysis (PLS-DA) displaying discriminant features. Shown are annotated metabolites in primed-unchallenged (*Pseudomonas fluorescens* N04) and primed-challenged (*Phytophthora capsici*, PC) plant tissues for root (R), stem (S) and leaf (L) tissues. Selected metabolites (coefficient scores ≥ 2) from primed-unchallenged plants (NT days 2 and 8) were compared to those in primed-challenged plants at the given time point (PC—2, 4, 6, 8 d.p.i.). The abbreviated names of the annotated metabolites are listed in Table S6.

Plants are known to modulate their metabolism in time-dependent responses to external stimuli [26,39]. Thus, to assess the metabolomic perturbations, chemometric analysis was used to investigate the dynamic metabolic defense differentiating PGPR-primed-unchallenged and PGPR-primed-challenged tomato plants. Here, PLS-DA was utilized to investigate metabolic reprogramming [41]. To determine the response of each feature to *Ph. capsici* infection, measurements of selected metabolites (VIP score ≥ 0.5) in infected plants were compared to those in control plants at the given time point and observed a differential metabolic reprogramming. The identified compounds spanned several metabolic pathways such as primary metabolism (amino acids and tricarboxylic acid cycle intermediates), fatty acids and secondary metabolites (phenylpropanoids, flavonoids, benzoic acids and glycoalkaloids) (Table S6). These molecules (discussed below) were found to accumulate in varying amounts in the different tissues and exhibit differential accumulation patterns over time upon challenge with *Ph. capsici* (Figure 6A,B). In general, these patterns indicate differential reprogramming over time (either high or low accumulation at specific time points, reflecting early-, late or oscillatory responses). The time-dependent reprogramming is an indication that plants fine-tune their defense response to better ward off infection.

## 3. Discussion

Harnessing plant–microbe interactions to promote disease resistance to pathogens could be a keystone in sustainable agriculture. PGPR priming/induced resistance is described as a systemic phenomenon triggered by these bacteria through interactions and chemical communication in the rhizosphere [50]. These interactions might render plants more effective in mounting a fast(er) and strong(er) defense response upon subsequent stresses [2,21]. This can be attributed to various physiological processes such as differential defense responses characterized by specific defense genes, proteins and metabolites.

Visually, plants subjected to PGPR priming exhibited no/reduced infection upon inoculation with *Ph. capsici* during the period 2–8 d.p.i. The results thus suggest that PGPR-inoculation leads to biochemical changes or perturbations within the plant tissues, phenotypically observable as induced resistance against *Ph. capsici*. Results from the quantitative estimation of the aromatic amino acids, Phe, Tyr and Trp, can be interpreted that the induction of ISR (especially in the case of *Pa. alvei* primed-challenged plants) involves the sensitization of the aromatic amino acid biosynthetic pathways. The rapid accumulation of aromatic amino acids indicates that pre-treatment with the PGPR pre-conditioned these pathways for enhanced and rapid accumulation upon *Ph. capsici* infection. Moreover, the observed differential perturbation by the two strains further suggests different mechanisms of action with regards to induction of ISR.

Functionally, the aromatic amino acids play a pivotal role in plant metabolism [51,52]. Besides being directly involved in protein synthesis, these amino acids serve as precursors for phytohormone synthesis (such a salicylic acid and auxins) and a wide range of secondary metabolites with an array of functions [52,53]. These amino acids are derived from chorismate, which is the initial branch point for the biosynthesis of various secondary metabolites and, as such, can be regarded as the regulatory link between primary and secondary metabolism in plants [51,53]. Moreover, in the Solanaceae family defense responses are mostly dominated by aromatic secondary metabolites, thus, the analysis of aromatic amino acids is essential to elucidate plant defense responses at a metabolic level [54,55]. Plant metabolism is dynamic and plant–microbe interactions (either beneficial or pathogenic) lead to modifications in both primary and secondary metabolism. Various studies have investigated metabolic perturbations in plants by microbes and different observations have been made where microorganisms either cause an increase or decrease in specific metabolites [56,57,58]. These differential modifications have been found to be microbe-specific (different microbes cause diverse metabolomic perturbations) and plant-specific. For example, in *Arabidopsis thaliana* plants infected with *Ps. syringae* DC3000, only aspartic acid was found to be reduced by infection, while the other amino acids were either not affected or higher when compared to control plants. Among the increased amino acids were aromatic amino acids [59]. *Pa. alvei* T22 was found to prime sorghum plants against *Colletotrichum sublineolum* infection and the primed state was characterized by a stronger accumulation of Trp and Tyr, as well as phenolic secondary metabolites derived from the phenylpropanoid pathway [26]. These findings correlate with the obtained results where an increase/accumulation of aromatic amino acids as a component of the priming mechanism against *Ph. capsici* was observed.

The differential regulation of ABA and MeSA content in PGPR-primed plants indicates priming to be highly dependent on the priming agent which may also be true for the response to secondary stresses. Moreover, the differential perturbation of the two phytohormones observed in response to the different PGPR treatments indicates that the plant defense responses are fine-tuned based on the perceived stimuli. Upon pathogen infection, plants generate and transport signaling molecules to surrounding cells and distal plant parts to induce a defense response [28,60]. Among these signal molecules (often referred to as phytohormones), MeSA and ABA showed differential regulation during pathogen progression, and were found to be above the limit of quantification for the different time points. The role of ABA in development, root geotropism, opening of stomata through stomatal guard cells and dormancy of buds has been extensively documented. Furthermore, ABA is involved in plant responses to wounding and pathogen infection [61,62,63]. On the other hand, MeSA is a volatile phytohormone released by plants upon pathogen infection or herbivore feeding [64]. When plants are under attack, MeSA is accumulated in the affected tissue and transported throughout the plant as well as to neighboring plants due to its volatility [65]. Once in the distal tissue or neighboring plants, MeSA is converted to SA in healthy tissue to prepare these for potential infection [66,67,68].

With regards to the chemometric analysis of the untargeted metabolomics data, both PCA and HiCA assisted in evaluating the overall structure of the data sets, revealing underlying patterns and trends (i.e., time- and treatment-dependent groupings). These observations highlight aspects of the biochemical phenomena (altered metabolomic states) attributed to PGPR priming against *Ph. capsici*. In general, these patterns indicate differential reprogramming over time (either high or low accumulation at specific time points, reflecting early-, late or oscillatory responses). The time-dependent reprogramming is an indication that plants utilize multiple interlinked metabolic pathways as part of their defense response to better ward off infection.

Besides being involved in plant growth and development, recent studies demonstrated that primary metabolism undergoes reprogramming during both priming and immune response activation [69]. Primary metabolites are the main source of energy required to initiate plant defense responses and provide precursors for the synthesis of signaling molecules and phytoalexins [2,70]. Using untargeted LC–MS to investigate the metabolic events associated with PGPR priming (*Ps. fluorescens* N04 and *Pa. alvei* T22), primary metabolites belonging to amino acids (Trp, Tyr, Phe, Pro and Acetyl trp) and the TCA cycle (two citric-acid isomers and malic-acid) were annotated. Moreover, sugar- conjugated molecules were also annotated (Table S6). PGPR (both *Ps. fluorescens* and *Pa. alvei*) primed-challenged plants exhibited a differential and time-dependent metabolic reprogramming in the various tissues (Figure 6). The aromatic amino acids serve as precursors for phenolics and other molecules involved in defense signaling and resistance. For instance, Phe is the precursor for the phenylpropanoid pathway leading to the production of hydroxycinnamic acid (HCA) derivatives and flavonoids [26,55], whereas Trp and its derivative (acetyl Trp) are involved in the biosynthesis of defense signaling molecules such as auxin and some phytoalexins such as serotonin conjugates [51,71,72]. The role of Pro in plant defense responses to pathogens is still not well understood, however, it has been reported to supply both carbon and nitrogen for the synthesis of defense-related metabolites [73].

Organic acids are precursors for the biosynthesis of different metabolites and here three TCA intermediates were identified. The TCA cycle is the central metabolic pathway for energy production from carbohydrates and fatty acid oxidation. Moreover, the TCA cycle leads to the production of fatty acids and here oxygenated octadecanoic acid derivatives (trihydroxyoctadecadienoic acid, C_18_H_32_O_5_ I, II, and trihydroxyoctadecenoic acid, C_18_H_34_O_5_ I, II) were annotated. These molecules were also differentially regulated in the various tissues (Figure 6). A role for fatty acids in plant defense can be related to lipid signaling or membrane disruption associated with the hypersensitive response [74].

Flavonoids are phenolic molecules derived from the phenylpropanoid pathway and play different roles in plant defense responses, such as in signaling, as antioxidants, and in biotic and abiotic resistance in general. These are naturally occurring in plant tissue, however, the content can be differentially reprogrammed during plant defense responses [56,58]. For example, the flavonoid content was found to be higher in potato plants infected with *Botrytis cinerea* and exhibited a decrease with disease progression [44]. Moreover, in PGPR-primed sorghum plants, flavonoids were reported as signatory biomarkers for priming against *C. sublineolum* [26] and *Fusarium pseudograminearum* [75] infection.

Hydroxycinnamic acids are widely distributed in plants and have been associated with priming [40,55] and defense responses [58]. Here, HCAs conjugated to sugars, polyamines and quinic acids were annotated as discriminatory biomarkers in PGPR-primed plants challenged by *Ph. capsici*. These molecules showed increasing or decreasing trend patterns, suggestive of increased biosynthesis followed by interconversion, incorporation into insoluble polymers and degradation (Figure 6). HCAs like ferulic, caffeic, p-coumaric and sinapic acids are functional antimicrobial compounds, and precursors to the synthesis of both inducible and constitutive defense metabolites. These are also key in structural defenses as monolignol precursors of lignin and by participating in cross-linking primary cell wall polysaccharides. HCA amides, such as 4-coumaroylagmatine and feruloylserotonin, are also known in the context of cell wall strengthening, as well as antimicrobial compounds [76].

Steroidal glycoalkaloids (SGAs) are nitrogen-containing compounds bearing a sugar chain (three or four units) linked to a steroidal moiety (aglycone) by the 3-hydroxyl group. SGAs are naturally occurring phytoanticipins and accumulation of these molecules throughout the different plant tissues can prevent both herbivore feeding and plant disease. Previous studies have demonstrated that SGAs have antimicrobial activities and are perturbed in response to pathogen infection [58,77]. However, microorganisms have evolved mechanisms that enable them to reduce SGAs toxicity (by secreting glycoside hydrolases) [44,78]. In the present study, SGAs were identified as part of the discriminatory biomarkers, and time-differential reprogramming was observed in the roots, stems and leaves of plants primed with *Ps. fluorescens* and *Pa. alvei* and challenged with *Ph. capsici* (Figure 6). These molecules were found to be either lower/higher at a specific time point (2, 4, 6, 8 d.p.i.) in primed plants responding to the pathogen.

Previous metabolic studies on A. thaliana plants [79] and tobacco cells [14,40] have demonstrated that different priming agents activate similar metabolic pathways, but where the resulting metabolomes differ with regards to the metabolic composition such as the presence/absence and quantity of specific metabolites. Similarly, sorghum plants responding to *Burkholderia andropogonis* [56] and *C. sublineolum* [57], were characterized by activation of similar metabolic pathways, “fine-tuned” in terms of quantity and composition. Moreover, where these plants were pre-conditioned by *Pa. alvei* followed by *C. sublineolum* infection [26] and *F. pseudograminearum* infection [75], comparable results were observed.

The tomato plants thus seem to mobilize similar defense responses (as reflected by activation of similar pathways leading to secondary metabolite synthesis) and at a metabolite level (e.g., enhanced synthesis of secondary metabolites with antimicrobial and anti-oxidant activities), but fine-tune the defense based on the perceived stimulus and the existing biochemical background operative in the naïve vs. primed conditions. Allowing for the dynamic nature of plant metabolism, qualitative and quantitative differences of specific metabolites or classes of metabolites within the broader metabolomic profiles, may modulate the eventual outcome of a host response to attempted infection.

## 4. Materials and Methods

### 4.1. Plant Material, PGPR Treatment and Infection with Phytophthora capsici

The two PGPR strains (*Pseudomonas fluorescens* N04 and *Paenibacillus alvei* T22), selected based on previous data where it has been reported that these strains enhance plant growth and induce resistance against biotic and abiotic stresses, were obtained from the culture collection of Prof. Nico Labuschagne, University of Pretoria, South Africa and stored and cultured as previously described [41]. Furthermore, *Ps. fluorescens* is one of the documented organisms in plant growth stimulation, while *Pa. alvei* has recently been found to induce resistance in sorghum plants against *C. sublineolum* [26] and *F. pseudograminearum* [75]. The virulent strain of *Ph. capsici* (PRRI 20101) used in this study was obtained from the Plant Protection Institute, Agricultural Research Council (ARC), Pretoria, South Africa [42]. Tomato (*Solanum lycopersicum* cv. Moneymaker) seeds were obtained from a commercial seed company, Starke Ayres (Bredell, South Africa).

### 4.2. Experimental Design

The contents of this report are related to two previously published studies. In the first paper [41], we reported on the effect of PGPR bacteria (*Ps. fluorescens* and *Pa. alvei*) on the metabolome of tomato plants and in the second paper [42], the host response of non-primed (naïve) tomato plants to the pathogen *Ph. capsisi* is described. For this study, the experimental design consisted of a control group, i.e., tomato plants pre-treated (primed) with the two PGPR-strains (harvested on days 2 and 8) and a treatment group, i.e., primed plants infected with *Ph. capsici* and harvested on days 2, 4, 6 and 8, following infection. The conditions on which the comparative metabolomics analysis was performed can thus be described as “primed-unchallenged” vs. “primed-challenged”.

Tomato seeds were germinated and grown in potted washed and autoclaved playpen sand. After 12 weeks, the plants were inoculated with the *Ps. fluorescens* and *Pa. alvei* as previously described [41]. Forty-eight h post-PGPR inoculation, the plants were stem-inoculated with 1 × 10^6^/mL spores of a virulent strain of *Ph. capsici* (PRRI 20101) [42]. During harvesting, the plants were separated into representative root, stem and leaf tissue samples, frozen in liquid nitrogen and stored at −80 °C.

The experimental design included three independent biological replicates that were each analyzed in triplicate, this generated *n* = 9 as required for metabolomic analysis.

### 4.3. Extraction and Quantification of Phytohormones and Aromatic Amino Acids by Multiple Reaction Monitoring Mass Spectrometry (MRM-MS)

Aromatic amino acids (Phe, Tyr and Trp) and phytohormones (indole acetic acid, abscisic acid, salicylic acid, jasmonic acid, 1-aminocyclo propane-1-carboxylic acid, methyl jasmonate and methylsalicylate) were extracted and quantified as previously described [41,42,43,46]. Briefly, 200 mg was extracted with 1 mL 50% ice-cold methanol (containing 1 ng/µL of the internal standard prednisolone) using 2 mL microcentrifuge “BashingBead” lysis tubes containing ceramic microbeads (Zymo Research, Irvine, CA, USA). The samples were further homogenized with a FastPrep FP120 instrument at 5 °C for 3 min at high speed, centrifuged at 13,000× *g* (Eppendorf microfuge, Merck, Modderfontein, South Africa) for 15 min at 5 °C and the supernatants transferred to 2 mL microcentrifuges tubes. The pellets were then re-extracted with 0.5 mL 50% ice-cold methanol. The two extracts were combined and filtered through a 0.22 µm nylon filter into 2 mL vials and stored at −20 °C until analyzed. The filtered 50% methanol extracts were analyzed on a Nerexa UHPLC (Shimadzu Corporation, Tokyo, Japan) fitted with a Restek Ultra AQ C18 column (100 mm × 2.1 mm × 3 µm), thermostatted at 40 °C. The UHPLC was coupled to a Shimadzu triple quadrupole mass spectrometer (QqQ-MS) (Model 8050, Shimadzu Corporation, Tokyo, Japan). Both chromatographic and MS parameters were as detailed for multiple reaction monitoring (MRM) MS [41]. Each MRM transition energy was optimized using “MRM optimization method tool” a component of the LabSolution LC-MS software (Shimadzu Corporation, Kyoto, Japan). MRM transitions, LOD and LOQ values can be found on Tables S2 and S3, respectively. Recovery values for ABA and MeSA can be found in Table S4. LC–MS data acquisition was carried out in triplicate and the results were expressed as mean values ± standard deviation (SD).

#### Statistical Analysis of Amino Acid and Phytohormone Data

Statistical analysis included univariate analysis of variance (ANOVA), performed as 2-tailed complete randomized blocks and used to compare the non-inoculated vs. PGPR inoculated plants at different time points [41]. ANOVA was followed by the Tukey post-hoc test where differences between the means were considered significant at *p* < 0.05, indicated in graphs with a dot (•) an asterisk (*) or an alphabet letter (a). The dot (•) indicates significant difference between the day 2 PGPR-primed-unchallenged (NT) control and day 2/4/6/8 PGPR-primed-challenged (PC) treatment groups. Similarly, an asterisk (*) indicates the significance of differences between the day 2/4/6/8 treatment groups but compared to the day 8 PGPR-primed-unchallenged (NT) control. The letter “a” indicates a statistically significant difference between two controls, NT day 2 and NT day 8. The summarized outputs are presented in Tables S1 and S5.

### 4.4. Metabolite Profiling: Sample Preparation, Analysis and Data Acquisition

Metabolite extraction and sample preparation from plants was carried out as previously described [41]. In short, the plant material was snap frozen in liquid nitrogen and ground with a motor and pestle. One gram (250 mg for roots) of the lyophilized samples was extracted with 10 mL of 80% methanol in a ratio of 1:10 (*w*/*v*) and sonicated for 20 min in a sonicator bath. Samples were then centrifuged, and the supernatants were concentrated to approximately 1 mL and dried overnight in a heating block. The resulting pellet was resuspended in 300 µL 80% methanol, filtered through a 0.22 µm nylon filter into a 2 mL vial fitted with 500 µL insert. Quality control (QC) samples were pooled and prepared by mixing equal volumes of all samples. Samples were then stored at −20 °C until analysis. Two µL of the extracts were analyzed on a Waters Acquity UHPLC coupled to a Waters SYNAPT G1 Q–TOF mass spectrometer (Waters Corporation, Milford, MA, USA). The extracts were chromatographically separated on a Waters HSS T3 C18 column (150 mm × 2.1 mm × 1.8 µm), with an oven temperature set at 60 °C. Both chromatographic—and MS conditions were as detailed [41]. Briefly, the run was 30 min, and the mobile phase composition was as follows: 0.1% aqueous formic acid and 2.5% isopropyl alcohol in water (Sigma-Aldrich, Munich, Germany) (solvent A), and 0.1% formic acid and 2.5% isopropyl alcohol in acetonitrile (Romil Pure Chemistry, Cambridge, UK) (solvent B) at a flow rate of 0.4 mL/min. The linear gradient was as follows: 0.00–1.00 min, 2% B; 1.0–22.00 min, 2–60% B; 22.00–23.00 min, 60–95% B; 23.00–26.00 min, 95% B; 26.00–27.00 min, 95–5% B and 27.00–30.00 min, 5% B. Samples were randomized prior to data acquisition, and the QC sample was used to assess the instrument reliability and reproducibility and injected every 10 injections. Each sample was analyzed in triplicate.

Analysis was performed in both negative and positive electrospray ionization (ESI) mode using a scan range from *m*/*z* 100–1500 Da. The MS conditions were capillary voltage: 2.0 kV; sample cone voltage: 40 V; microchannel plate (MCP) detector voltage: 1600 V; source temperature: 120 °C; and desolvation temperature: 450 °C; cone gas flow rate was 50 L/h and the desolvation gas flow 550 L/h; scan time: 0.10 s; interscan delay: 0.02 s; mode: centroid. Lastly, the MS was set to acquire both unfragmented and five fragmenting experiments (MS^E^) simultaneously by ramping in-source collision energy from 3 eV to 30 eV.

### 4.5. Statistical Analyses of Multivariate Data (MVDA)

The methodology described in [41,42] was adopted for this section. Briefly, MarkerLynx XS^TM^ software (Waters Corporation, Manchester, UK) was used for data pre-processing, producing a matrix of retention time (Rt), and mass to charge ratio (*m*/*z*) with peak intensities of each sample. The matrices were created using the following parameters: Rt range 1.0–25 min, Rt difference 0.2 min, *m*/*z* range 50–1500, *m*/*z* difference 0.05, mass tolerance 0.5, intensity threshold count 15 and noise level 5. It is important to note that these parameters were adapted based on visual inspection of the MS chromatograms (Figures S1–S3). The generated matrices were exported to SIMCA (soft independent modelling by class analogy) software, version 15 (Sartorius Stedim Data Analytics AB, Umeå, Sweden) for chemometric analysis. Data were Pareto-scaled prior to analysis. LC–MS metabolic profiling consists of thousands of features and is multidimensional. To reduce this complexity of the data sets, two unsupervised methods namely principal component analysis (PCA) and hierarchical clustering analysis (HiCA) were applied for MVDA.

Statistical evaluation of the PCA clustering and HiCA tree is reported in the legends to the respective figures. In addition, a supervised method, orthogonal projection to latent structures discriminant analysis (OPLS-DA) was used. OPLS-DA models were statistically validated using cross-validated analysis of variance (CV-ANOVA) with *p* ≤ 0.05 indicating a good model, using the inbuilt SIMCA seven-fold (default) CV method and with a permutation test of 10 permutations. Subsequent interpretation was assisted by OPLS-DA S-plots, where significant ions with the correlation (*p*(*corr*)) ≥ 0.6 and covariance of (*p1*) ≥ 0.05 were selected for exploration and annotation as illustrated in Figure 5. For OPLS-DA analyses, the primed condition on day 8 was compared to the primed-infected condition on day 6 so as to relate to the naïve (infection only) plant study where data from day 6 of *Ph. capsici*-infected plants were chosen for analysis since the plants were in an advanced stage of death on day 8 [42]. OPLS-DA models of analyzed data from extracts obtained from roots, stems and leaves are also presented as Figures S9–S13.

In addition, MetaboAnalyst 5.0 (www.metaboanalyst.ca, accessed on 21 August 2020)—online processing of metabolomics data for statistical, functional and integrative analysis—for multivariate statistical analyses [80,81,82] was used for partial least square-discriminant analysis (PLS-DA) (a supervised method) and applied to investigate time-dependent metabolic reprogramming in primed-unchallenged and primed-challenged plant tissues for root, stem and leaf tissues.

### 4.6. Metabolite Annotation

Data were acquired using five different collision energies (MS^E^), ramping from 0 to 50 eV to cause fragmentation of the initial ions so as to ensure that as much information regarding the structures of the respective compounds could be obtained for downstream structural elucidation and metabolite annotation. Accurate mass, empirical formulae generation and MS-based compound annotation (at a metabolite identification (MI) level-2 annotation) were as detailed [41,42].

## 5. Conclusions

Plant immune receptors and pathogen-derived molecules are part of an intricate sensing and multi-layered signaling network comprising the plant innate immune system. Recognition events (either at the cell surface or intracellularly) results in activation of defense responses that manifests as changes to the metabolome of the infected plant. Targeted LC–MS analysis showed a time-dependent regulation of the amino acids Phe, Trp and Tyr, and the phytohormones MeSA and ABA as part of the response of primed tomato host plants to infection by *Ph. capsici*. The aromatic amino acid content was found to undergo differential reprogramming in plants pre-treated with *Ps. fluorescens* and *Pa. alvei* and subsequently inoculated with to *Ph. capsici*. The metabolic response of the latter challenged plants (*Pa. alvei*/*Ph. capsici*) were characterized by a rapid and intense accumulation of the aromatic amino acids when compared to primed-unchallenged plants (*Pa. alvei* only). By comparison, *Ps. fluorescens* primed-challenged plants showed a slow accumulation of the amino acids compared to *Ps. fluorescens* primed-unchallenged plants. More-over, ABA and MeSA were two phytohormones found to be upregulated signaling molecules in the primed plants in response to *Ph. capsici*. These metabolites were found to be differentially regulated in plants primed by both *Ps. fluorescens* and *Pa. alvei,* responding to *Ph. capsici* infection. Furthermore, in the various tissues, the levels of these phytohormones were either tightly regulated with minor changes, except for MeSA which accumulated in higher concentrations in the various tissue of *Ps. fluorescens* primed-challenged plants. On the other hand, *Pa. alvei* primed-challenged plants showed a decrease in MeSA content in root tissue and no or little change in the stem tissue, while an increase over time was observed in leaf tissue.

In systems biology metabolomics, the aim is to investigate the interaction of as many as possible metabolites within a system in order to understand how the system operates under some predetermined perturbation. Here, untargeted LC–MS metabolomics analysis was used to decode the signaling and defense responses of tomato plants under biotic stress. The results revealed time-dependent metabolic changes in the PGPR-primed-unchallenged vs. PGPR-primed-challenged plant tissues. The annotated metabolites included amino acids and TCA cycle organic acids, as well as hydroxylated fatty acids as primary metabolites and phenylpropanoids, benzoic acids, flavonoids and glycoalkaloids as secondary metabolites. Tissue-specific reprogramming was also observed, which demonstrated that the root, stem and leaf tissues of the PGPR-treated plants undergo differential metabolomic changes in response to subsequent infection. Superficially distinct metabolic pathways may show a level of interconnectivity and feedback loops that allow rapid responses to external stimuli and cellular injury. These perturbations can thus be interpreted as being due to the activation or mobilization of chemical defenses against the pathogen in an attempt to ward off the infection or limit the cellular damages caused by the infection.

Overall, the results show that the tomato plants are genetically enabled to effect internal metabolic changes in order to adapt to changing environmental conditions (e.g., in establishing the primed condition) and external stimuli such as pathogen attack by *Ph. capsici*. The annotated metabolites and pathways were interconnected with each other, unveiling a pivotal metabolic network associated with the tomato host response to *Ph. capsici*. While the relative importance of the individual discriminatory features as potential bio-markers) cannot be judged from the available data, the identified metabolic pathways to which they belong could pave the way for further studies in elucidating PGPR priming mechanisms and their potential use in the agricultural industry.

## Data Availability

The study design information, LC-MS data, data processing and analyses are reported on and incorporated into the main text. Raw data, analyses and data processing information, and the meta-data are deposited to the EMBL-EBI metabolomics repository—MetaboLights50, with the identifier MTBLS2904 (http://www.ebi.ac.uk/metabolights/MTBLS2904, accessed on 21 August 2020).

## Supplementary Materials

The following are available online at https://www.mdpi.com/article/10.3390/plants10081530/s1, Table S1. One-way ANOVA comparing mean values of quantified aromatic amino acids and phytohormones in PGPR-primed-unchallenged vs. PGPR-primed-challenged tomato plant tissues. Table S2. Annotation of individual internal standard, amino acids and phytohormones by retention time, *m*/*z* values and identification by MS/MS fragmentation patterns. Table S3. Parameters of the calibration curves for each amino acid and phytohormone: analytical range, regression, limit of detection (LOD) and limit of quantification (LOQ). Table S4. Percentage of recovery during phytohormone extraction from tomato plant tissue. Table S5. Post-hoc tests comparing mean values of quantified aromatic amino acids and phytohormones in PGPR-primed-unchallenged vs. PGPR-primed-challenged tomato plant tissues. Table S6. Summary of annotated (MSI-level 2) metabolites that contributed to the discriminating variability in the altered metabolomes (as described by chemometric models). Figure S1. Representative BPI MS chromatograms of extracts from leaf tissue from primed-unchallenged (*Paenibacillus alvei* T22 NT), and primed-challenged (*Paenibacillus alvei* T22 PC) tomato plants. Figure S2. Representative BPI MS chromatograms of extracts from stem tissue from primed-unchallenged (*Paenibacillus alvei* T22 NT) and primed-challenged (*Paenibacillus alvei* T22 PC) tomato plants. Figure S3. Representative BPI MS chromatograms of extracts from root tissue from primed-unchallenged (*Paenibacillus alvei* T22 NT) and primed-challenged (*Paenibacillus alvei* T22 PC) tomato plants. Figure S4. Unsupervised statistical analysis of tomato leaf data acquired in ESI^+^ mode comparing primed by *Pa. alvei* T22 and primed-challenged plants. Figure S5. Unsupervised statistical analysis of tomato stem data acquired in ESI^−^ mode comparing primed by *Pa. alvei* T22 and primed-challenged plants. Figure S6. Unsupervised statistical analysis of tomato stem data acquired in ESI^+^ mode comparing primed by *Pa. alvei* T22 and primed-challenged plants. Figure S7. Unsupervised statistical analysis of tomato root data acquired in ESI^−^ mode comparing primed by *Pa. alvei* T22 and primed-challenged plants. Figure S8. Unsupervised statistical analysis of tomato root data acquired in ESI^+^ mode comparing primed by *Pa. alvei* T22 and primed-challenged plants. Figure S9. OPLS-DA modeling and variable/feature selection of leaf data acquired in ESI^+^ mode comparing primed (*Paenibacillus alvei* T22 NT) and primed-challenged (*Paenibacillus alvei* T22 PC) plants. Figure S10. OPLS-DA modeling and variable/feature selection of stem data acquired in ESI^−^ mode comparing primed (*Paenibacillus alvei* T22 NT) and primed-challenged (*Paenibacillus alvei* T22 PC) plants. Figure S11. OPLS-DA modeling and variable/feature selection of stem data acquired in ESI^+^ mode comparing primed (*Paenibacillus alvei* T22 NT) and primed-challenged (*Paenibacillus alvei* T22 PC) plants. Figure S12. OPLS-DA modeling and variable/feature selection of root data acquired in ESI^−^ mode comparing primed (*Paenibacillus alvei* T22 NT) and primed-challenged (*Paenibacillus alvei* T22 PC) plants. Figure S13. OPLS-DA modeling and variable/feature selection of root data acquired in ESI^+^ mode comparing primed (*Paenibacillus alvei* T22 NT) and primed-challenged (*Paenibacillus alvei* T22 PC) plants.
