# Supplementary material for: Metabolomic Evaluation of Tissue-Specific Defense Responses in Tomato Plants Modulated by PGPR-Priming against Phytophthora capsici Infection
Source: Plants (Basel). 2021 Jul 26;10(8):1530. doi: 10.3390/plants10081530 (PMC8400099; doi:10.3390/plants10081530)
Supplement: Supplementary file 1 [file plants-10-01530-s001.zip › plants-1303998-supplementary.pdf]

plants

Metabolomic evaluation of tissue-specific defense responses modulated by PGPR-treatment against *Phytophthora capsici* in tomato plants

Msizi I. Mhlongo[1], Lizelle A. Piater[1], Paul A. Steenkamp[1] Nico Labuschagne[2] and Ian A. Dubery[1]*

[1] Research Centre for Plant Metabolomics, Department of Biochemistry, University of Johannesburg, Auckland Park, 2006, Johannesburg, South Africa; [2] Department of Plant and Soil Sciences, University of Pretoria, 0028, South Africa.
*Correspondence: E-mail: idubery@uj.ac.za; Tel.: +27-11-559-2401.

Supplementary Files –

Table S1. One-way ANOVA comparing mean values of quantified aromatic amino acids and phytohormones in PGPR-primed-unchallenged *vs.* PGPR-primed-challenged tomato plant tissues.

Table S2. Annotation of individual internal standard, amino acids and phytohormones by retention time, *m*/*z* values and identification by MS/MS fragmentation patterns.

Table S3. Parameters of the calibration curves for each amino acid and phytohormone: analytical range, regression, limit of detection (LOD) and limit of quantification (LOQ).

Table S4. Percentage of recovery of abscisic acid and methyl salicylate during phytohormone extraction from tomato plant tissue.

Table S5. Post-hoc tests comparing mean values of quantified aromatic amino acids and phytohormones in PGRP-primed-unchallenged *vs.* PGPR-primed-challenged tomato plant tissues.

Table S6. Summary of annotated (MSI-level 2) metabolites that contributed to the discriminating variability in the altered metabolomes (as described by chemometric models).

Figure S1. Representative BPI MS chromatograms of extracts from leaf tissue from primed-unchallenged (*Paenibacillus alvei* T22 NT), and primed-challenged (*Paenibacillus alvei* T22 PC) tomato plants.

Figure S2. Representative BPI MS chromatograms of extracts from stem tissue from primed-unchallenged (*Paenibacillus alvei* T22 NT) and primed-challenged (*Paenibacillus alvei* T22 PC) tomato plants.

Figure S3. Representative BPI MS chromatograms of extracts from root tissue from primed-unchallenged (*Paenibacillus alvei* T22 NT) and primed-challenged (*Paenibacillus alvei* T22 PC) tomato plants.

Figure S4. Unsupervised statistical analysis of tomato leaf data acquired in ESI^+ mode comparing primed by *Pa. alvei* T22 and primed-challenged plants.

Figure S5. Unsupervised statistical analysis of tomato stem data acquired in ESI^- mode comparing primed by *Pa. alvei* T22 and primed-challenged plants.

Figure S6. Unsupervised statistical analysis of tomato stem data acquired in ESI^+ mode comparing primed by *Pa. alvei* T22 and primed-challenged plants.

Figure S7. Unsupervised statistical analysis of tomato root data acquired in ESI^- mode comparing primed by *Pa. alvei* T22 and primed-challenged plants.

Figure S8. Unsupervised statistical analysis of tomato root data acquired in ESI^+ mode comparing primed by *Pa. alvei* T22 and primed-challenged plants.

Figure S9. OPLS-DA modeling and variable/feature selection of leaf data acquired in ESI^+ mode comparing primed (*Paenibacillus alvei* T22 NT) and primed-challenged (*Paenibacillus alvei* T22 PC) plants.

Figure S10. OPLS-DA modeling and variable/feature selection of stem data acquired in ESI^- mode comparing primed (*Paenibacillus alvei* T22 NT) and primed-challenged (*Paenibacillus alvei* T22 PC) plants.

Figure S11. OPLS-DA modeling and variable/feature selection of stem data acquired in ESI^+ mode comparing primed (*Paenibacillus alvei* T22 NT) and primed-challenged (*Paenibacillus alvei* T22 PC) plants.

Figure S12. OPLS-DA modeling and variable/feature selection of root data acquired in ESI^- mode comparing primed (*Paenibacillus alvei* T22 NT) and primed-challenged (*Paenibacillus alvei* T22 PC) plants.

Figure S13. OPLS-DA modeling and variable/feature selection of root data acquired in ESI^+ mode comparing primed (*Paenibacillus alvei* T22 NT) and primed-challenged (*Paenibacillus alvei* T22 PC) plants.

Table S1. One-way ANOVA comparing mean values of quantified aromatic amino acids and phytohormones in PGRP-primed-unchallenged *vs.* PGPR-primed-challenged tomato plant tissues.

Compound name	N04-primed-unchallenged *vs.* N04-primed-challenged *p*-value	T22-primed-unchallenged *vs.* T22-primed-challenged *p*-value
**Roots**		
Phe	0.000	0.000
Trp	0.000	0.000
Tyr	0.000	0.000
ABA	0.225	0.001
MeSA	0.000	0.000
**Stems**		
Phe	0.000	0.000
Trp	0.000	0.000
Tyr	0.000	0.000
ABA	0.000	0.000
MeSA	0.002	0.000
**Leaves**		
Phe	0.000	0.000
Trp	0.000	0.000
Tyr	0.000	0.000
ABA	0.001	0.001
MeSA	0.000	0.000

Table S2. Annotation of individual internal standard, amino acids and phytohormones by retention time, m/z values and identification by MS/MS fragmentation patterns. LC-ESI-MS/MS analyses were performed on a UHPLC system coupled to a triple quadrupole mass spectrometer operating in positive ion mode.

No.	Analyte	Pseudo-molecular ions	Rt (min)	*m/z*	Fragment ions (*m/z*) observed in MRM mode and relative intensity (%)	Quadrupole 1 (Q1), V	Collision energy (CE), V
**1**	Pred	$[M+H]^+$	8.30	361.00	361.00 > 343.25	-20.00	-10.00
					361.00 > 325.20	-20.00	-11.00
					361.00 > 147.15	-19.00	-22.00
**2**	Phe	$[M+H]^+$	1.11	166.00	166.00 > 120.20	-12.00	-15.00
					166.00 > 103.15	-12.00	-28.00
					166.00 > 77.15	-12.00	-40.00
**3**	Trp	$[M+H]^+$	1.43	205.05	205.05 > 188.20	-10.00	-12.00
					205.05 > 146.15	-10.00	-19.00

					205.05 > 118.20	-10.00	-26.00
**4**	Tyr	$[M+H]^+$	0.80	182.05	182.05 > 91.20	-13.00	-30.00
					182.05 > 136.20	-13.00	-16.00
					182.05 > 165.15	-10.00	-13.00
**5**	ABA	$[M+H]^+$	7.90	265.10	265.10 > 247.20	-20.00	-8.00
					265.10 > 229.30	-13.00	-10.00
					265.10 > 201.15	-13.00	-13.00
**6**	**MeSA**	$[M+H]^+$	8.95	153.00	153.00 > 121.20	-16.00	-19.00
					153.00 > 65.15	-11.00	-29.00
					153.00 > 93.05	-11.00	-24.00

Table S3. Parameters of the calibration curves for each amino acid and phytohormone: analytical range, regression, limit of detection (LOD) and limit of quantification (LOQ).

Compound	Range (ng/µL FW)	Curve	R^2	LOD (ng/µL)	LOQ (ng/µL)
**Pred**	0.0001 – 20	$Y = 1.59^6X + 717\ 037$	99.54	> 0.001	0.001
**Phe**	0.0001 – 20	$Y = 7.98^6X + 1.16^6$	99.50	> 0.0001	0.0001
**Trp**	0.0001 – 20	$Y = 8.21^6X + 4.52^6$	99.10	> 0.0001	0.0001
**Tyr**	0.0001 – 20	$Y = 520\ 669X + 411\ 370$	97.20	> 0.0001	0.0001
**ABA**	0.01 – 20	$Y = 1.29^6X + 103\ 687$	99.50	> 0.01	0.01
**MeSA**	0.1 – 20	$Y = 936\ 107X + 41\ 425$	99.86	> 0.1	0.1

LOD = the value corresponding to signal to noise ratio (S/N) = 3, while for LOQ is S/N = 10.

Table S4. Percentage of recovery of abscisic acid and methyl salicylate during phytohormone extraction from tomato plant tissue.

Compound	% of recovery (Mean)			
	**Low (1 ng/µL for ABA) (3 ng/µL MeSA)**	**Medium (6 ng/µL)**	**High (12 ng/µL)**	**Overall recovery (% average)**
**ABA**	89.21	85.89	76.98	87.03
**MeSA**	87.52	85.05	86.71	86.43

Table S5. Post-hoc tests comparing mean values of quantified aromatic amino acids and phytohormones in PGRP-primed-unchallenged *vs.* PGPR-primed-challenged tomato plant tissues.

Dependent Variable	(I) Condition	(J) Condition	N04 *p*-value	T22 *p*-value
**Roots**				
Phe	R NT Day 2	R NT Day 8	0.184	0.950
		R PC Day 2	0.000	0.000
		R PC Day 4	0.000	0.000
		R PC Day 6	0.000	0.000
		R PC Day 8	0.000	0.000
	R NT Day 8	R NT Day 2	0.184	0.950
		R PC Day 2	0.114	0.000
		R PC Day 4	0.000	0.000
		R PC Day 6	0.000	0.000
		R PC Day 8	0.000	0.000
Trp	R NT Day 2	R NT Day 8	1.000	0.841
		R PC Day 2	0.000	0.000
		R PC Day 4	0.000	0.000

		R PC Day 6	0.000	0.000
		R PC Day 8	0.000	0.000
	R NT Day 8	R NT Day 2	1.000	0.841
		R PC Day 2	0.000	0.000
		R PC Day 4	0.000	0.000
		R PC Day 6	0.000	0.000
		R PC Day 8	0.000	0.000
Tyr	R NT Day 2	R NT Day 8	0.000	0.999
		R PC Day 2	0.000	0.000
		R PC Day 4	0.000	0.000
		R PC Day 6	0.000	0.000
		R PC Day 8	0.000	0.000
	R NT Day 8	R NT Day 2	0.000	0.999
		R PC Day 2	0.000	0.000
		R PC Day 4	0.000	0.000
		R PC Day 6	0.000	0.000
		R PC Day 8	0.000	0.000
ABA	R NT Day 2	R NT Day 8	0.803	0.996
		R PC Day 2	0.998	1.000
		R PC Day 4	0.742	0.766
		R PC Day 6	1.000	0.862
		R PC Day 8	1.000	0.001
	R NT Day 8	R NT Day 2	0.803	0.996
		R PC Day 2	0.534	0.999
		R PC Day 4	0.108	0.961
		R PC Day 6	0.779	0.988
		R PC Day 8	0.729	0.006
MeSA	R NT Day 2	R NT Day 8	0.316	0.000
		R PC Day 2	0.017	0.000
		R PC Day 4	0.071	0.000
		R PC Day 6	0.154	0.000
		R PC Day 8	1.000	0.000
	R NT Day 8	R NT Day 2	0.316	0.000
		R PC Day 2	0.781	0.765
		R PC Day 4	0.975	0.470
		R PC Day 6	0.999	0.075
		R PC Day 8	0.193	0.288
**Stems**				
Phe	S NT Day 2	S NT Day 8	0.003	0.999
		S PC Day 2	1.000	0.000
		S PC Day 4	0.000	0.000
		S PC Day 6	0.000	0.000
		S PC Day 8	0.001	0.000
	S NT Day 8	S NT Day 2	0.003	0.999
		S PC Day 2	0.004	0.000
		S PC Day 4	0.000	0.000
		S PC Day 6	0.000	0.000
		S PC Day 8	0.000	0.000
Trp	S NT Day 2	S NT Day 8	0.000	0.994
		S PC Day 2	0.218	0.000
		S PC Day 4	0.000	0.000
		S PC Day 6	0.000	0.000
		S PC Day 8	0.000	0.000
	S NT Day 8	S NT Day 2	0.000	0.994
		S PC Day 2	0.000	0.000
		S PC Day 4	0.000	0.000
		S PC Day 6	0.000	0.000
		S PC Day 8	0.000	0.000
Tyr	S NT Day 2	S NT Day 8	0.000	0.956
		S PC Day 2	0.000	0.000
		S PC Day 4	0.088	0.000
		S PC Day 6	0.738	0.000
		S PC Day 8	0.003	0.000
	S NT Day 8	S NT Day 2	0.000	0.956

		S PC Day 2	0.000	0.000
		S PC Day 4	0.000	0.000
		S PC Day 6	0.000	0.000
		S PC Day 8	0.000	0.000
ABA	S NT Day 2	S NT Day 8	0.731	0.000
		S PC Day 2	0.157	0.002
		S PC Day 4	0.125	0.028
		S PC Day 6	0.000	0.983
		S PC Day 8	0.000	1.000
	S NT Day 8	S NT Day 2	0.731	0.000
		S PC Day 2	0.892	0.991
		S PC Day 4	0.846	0.682
		S PC Day 6	0.000	0.003
		S PC Day 8	0.000	0.000
MeSA	S NT Day 2	S NT Day 8	1.000	0.173
		S PC Day 2	0.151	0.202
		S4 PC Day 4	0.002	0.000
		S PC Day 6	0.199	0.000
		S PC Day 8	0.013	0.000
	S NT Day 8	S NT Day 2	1.000	0.173
		S PC Day 2	0.272	0.000
		S PC Day 4	0.004	0.019
		S PC Day 6	0.343	0.007
		S PC Day 8	0.030	0.215
**Leaves**				
Phe	L NT Day 2	L NT Day 8	0.000	0.927
		L PC Day 2	0.000	0.000
		L PC Day 4	0.000	0.000
		L PC Day 6	0.000	0.000
		L PC Day 8	1.000	0.000
	L NT Day 8	L NT Day 2	0.000	0.927
		L PC Day 2	0.000	0.000
		L PC Day 4	0.000	0.000
		L PC Day 6	0.000	0.000
		L PC Day 8	0.000	0.000
Trp	L NT Day 2	L NT Day 8	0.000	0.000
		L PC Day 2	0.004	0.000
		L PC Day 4	0.000	0.000
		L PC Day 6	0.000	0.000
		L PC Day 8	0.002	0.000
	L NT Day 8	L NT Day 2	0.000	0.000
		L PC Day 2	0.130	0.000
		L PC Day 4	0.000	0.000
		L PC Day 6	0.000	0.000
		L PC Day 8	0.132	0.000
Tyr	L NT Day 2	L NT Day 8	0.000	0.988
		L PC Day 2	0.000	0.000
		L PC Day 4	0.000	0.000
		L PC Day 6	0.240	0.000
		L PC Day 8	0.000	0.000
	L NT Day 8	L NT Day 2	0.000	0.988
		L PC Day 2	0.000	0.000
		L PC Day 4	0.000	0.000
		L PC Day 6	0.000	0.000
		L PC Day 8	0.764	0.000
ABA	L NT Day 2	L NT Day 8	0.641	0.961
		L PC Day 2	1.000	0.947
		L PC Day 4	0.277	0.001
		L PC Day 6	0.104	0.333
		L PC Day 8	0.472	0.999
	L NT Day 8	L NT Day 2	0.641	0.961
		L PC Day 2	0.774	1.000
		L PC Day 4	0.007	0.009
		LN04 PC Day 6	0.002	0.826

		LN04 PC Day 8	0.021	0.998
MeSA	L NT Day 2	LN04 NT Day 8	0.999	0.000
		LN04 PC Day 2	0.423	0.000
		LN04 PC Day 4	0.001	0.000
		LN04 PC Day 6	0.000	0.000
		LN04 PC Day 8	0.000	0.000
	L NT Day 8	LN04 NT Day 2	0.999	0.000
		LN04 PC Day 2	0.252	0.468
		LN04 PC Day 4	0.000	0.056
		LN04 PC Day 6	0.000	0.096
		LN04 PC Day 8	0.000	0.286

Table S6. Summary of annotated (MSI-level 2) metabolites that contributed to the discriminating variability in the altered metabolomes (as described by chemometric models). These discriminating metabolites were identified based on OPLS-DA S-plots, with a rigorous statistical validation (as explained in the text – Figure 6). These reported metabolites had VIP scores > 1.0.

No.	Rt (min)	Ionization mode	*m/z*	Compound Name	Abbreviation	MS fragments (*m/z*)
1	1.53	$[M-H]^-$	191.018	Citric acid I	C-acid I	173, 115, 111
2	1.53	$[M-H]^-$	341.106	Caffeoylglycoside	CGA	179
3	1.61	$[M+H]^+$	116.064	Proline	Pro	70
4	1.62	$[M-H]^-$	191.051	Quinic acid	Q-acid	173
5	1.75	$[M-H]^-$	133.009	Malic acid	M-acid	114, 89, 72
6	1.98	$[M-H]^-$	371.06	Caffeoylglucarate I	CGA	209, 191, 85
7	1.99	$[M-H]^-$	191.013	Citric acid II	C-acid II	173, 115, 111
8	2.12	$[M-H]^-$	191.014	3-Caffeoylquinic acid	3-CQA	191, 179, 135
9	2.12	$[M+H]^+$	182.075	Tyrosine	Tyr	91
10	2.28	$[M-H]^-$	371.059	Caffeoylglucarate II	CGA	209, 191, 85
11	2.74	$[M-H]^-$	371.057	Caffeoylglucarate III	CGA	209, 191, 85
12	3	$[M-H]^-$	203.076	Tryptophan	Trp	142, 116
13	3.01	$[M-H]^-$	371.058	Caffeoylglucarate IV	CGA	209, 191, 85
14	3.03	$[M-H]^-$	369.042	Sinapaldehyde glycoside	S-al glyc	147
15	3.38	$[M+H]^+$	166.079	Phenylalanine	Phe	120, 103, 91, 77
16	3.58	$[M-H]^-$	369.042	Sinapaldehyde glucoside	S-al glyc	147
17	3.61	$[M+H]^+$	251.134	Caffeoylputrescine	C-putr	234, 163, 145,135, 117, 89
18	4.01	$[M-H]^-$	353.082	4-CQA	3-CQA	191, 179, 173, 135
19	4.05	$[M-H]^-$	369.042	Sinapaldehyde glucoside	S-al glyc	147
20	4.19	$[M-H]^-$	353.084	5-Caffeoylquinic acid	5-CQA	191
22	4.58	$[M-H]^-$	285.057	Dihydroxybenzoic acid pentose	diHydro-Be acid pent	153
24	5.57	$[M-H]^-$	367.0245	3-Feruloylquinic acid	3-FQA	191
25	5.64	[M-H]	707.183	5-CQA	5-CQA	191
26	6.1	[M-H]	367.099	5-Feruloyquinic acid	5- FQA	191
27	6.46	[M-H]	385	Sinapoylglycoside	S-glyc	205
28	6.47	[M-H]	355.101	Feruloylglycoside	F-glyc	193
29	6.56	[M-H]	385.111	Sinapoylglycoside	S-glyc	203
30	6.62	[M-H]	385.107	Sinapoylglycoside	S-glyc	203
31	6.72	[M-H]	355.099	Feruloylglycoside	F-glyc	193
32	6.77	[M-H]	335.169	Caffeoylshikimic acid	CSA	179, 135
34	6.88	[M-H]	441.196	Benzyl alcohol dihexose	Ben-alc di-hex	269, 223, 161, 113, 101
35	7.63	[M-H]	411.184	Caffeoylputrescine glyco-side	C-putr glyc	321, 249, 135
36	7.66	[M-H]	367.092	5-Feruloylquinic acid	5-FQA	191
37	7.72	[M-H]	609.146	Rutin	Rutin	300
38	8.36	[M-H]	741.195	Quercetin-3-O-trisacha-ride	Qu-3-O-tri-sach	300
39	8.95	$[M+H]^+$	412.212	Tomatid-4-en-3-one	Tomat-one	325, 271, 161
42	9.58	[M-H]	245.087	Acetyl tryptophan	Acetyl trp	203

43	9.66	[M-H]	474.176	Feruloyltyramine glycoside	F-tyr glyc	312, 178
44	11.02	$[M+H]^+$	1050.28	Neorickiioside A	Neo A	1032, 414, 273, 255,
45	11.21	$[M+H]^+$	1050.213	Lycoperoside H	Lyc H	1032, 576, 272, 255
46	11.24	$[M+H]^+$	1032.56	Dehydrotomatine	De-tom I	576, 558, 414
47	11.64	$[M+H]^+$	1032.56	Dehydrotomatine	De-tom II	576, 558, 414
48	11.89	$[M+H]^+$	1032.56	Dehydrotomatine	De-tom III	576, 558, 414
49	12.23	$[M+H]^+$	1032.56	Dehydrotomatine	De-tom IV	576, 558, 414
50	12.57	$[M+H]^+$	1034.57	α-Tomatine I	α-Tom I	578, 560, 416, 255, 161
51	12.62	$[M+H]^+$	1032.56	Dehydrotomatine	De-tom	576, 558, 414
52	12.87	$[M+H]^+$	1034.57	*α*-Tomatine	α-Tom II	578, 560, 416, 255, 161
53	12.91	$[M+H]^+$	1032.56	Dehydrotomatine	De-tom	576, 558, 414
54	13.27	$[M+H]^+$	295.096	*α*-Tomatine	α-Tom III	578, 416
55	13.33	$[M+H]^+$	1034.25	*α*-Tomatine	α-Tom IV	578, 416
56	13.35	$[M+H]^+$	1004.56	Tomatidine dihexoside dipentoside	Tomati dihex dipent	569
57	13.55	$[M+H]^+$	1034.57	*α* –Tomatine	α-Tom V	578, 560, 416, 255, 161
58	14.04	$[M+H]^+$	1240.64	*α*-Tomatine	α-Tom VI	578, 560, 416, 255, 161
59	14.36	$[M+H]^+$	414.331	Tomatodenol +232	Tomato conj	273, 255, 161
60	14.38	$[M-H]^-$	447.221	Kaempferol-3-glucoside	Ka-3-gluc	285
61	15.15	$[M+H]^+$	414.332	Tomatodenol + 248	Tomato conj	273, 255, 161
62	15.76	$[M-H]^-$	327.213	Trihydroxy-octadecadienoic acid I	$C_{18}H_{32}O_5$	171
63	15.96	$[M+H]^+$	412.315	Tomatid-4-en-3-one	Tomat-one	351, 325, 271
64	16.06	$[M-H]^-$	327.213	Trihydroxy-octadecadienoic acid II	$C_{18}H_{32}O_5$	171
65	16.41	$[M+H]^+$	414.33	Tomatodenol	Tomato I	273, 255, 161
66	16.77	$[M-H]^-$	329.229	Trihydroxy-octadecenoic acid I	$C_{18}H_{34}O_5$	271, 171, 139
67	16.8	$[M-H]^-$	327.212	Trihydroxy-octadecadienoic acid III	$C_{18}H_{32}O_5$	171
68	16.96	$[M+H]^+$	416.348	Tomatidine	Tomati I	273, 255, 163
69	17.05	$[M-H]^-$	329.228	Trihydroxy-octadecenoic acid II	$C_{18}H_{34}O_5$	171, 139
70	17.06	$[M+H]^+$	414.333	Tomatodenol	Tomato II	273, 255, 161
71	17.83	$[M+H]^+$	414.332	Tomatodenol	Tomato III	273, 255, 161
72	17.85	$[M+H]^+$	416.349	Tomatidine	Tomati II	273, 255, 163
73	18.75	$[M+H]^+$	414.333	Tomatodenol	Tomato IV	273, 255, 161
75	19.87	$[M-H]^-$	593.281	Kaempferol-O-B-rutinoside	Ka-3-O-*B*-rut	285
78	21.73	$[M-H]^-$	593.275	Kaempferol-O-B-rutinoside	Ka-3-O-*B*-rut	285
79	22.65	$[M-H]^-$	474.26	Feruloytyramine glycoside	F-tyr glyc	312, 178

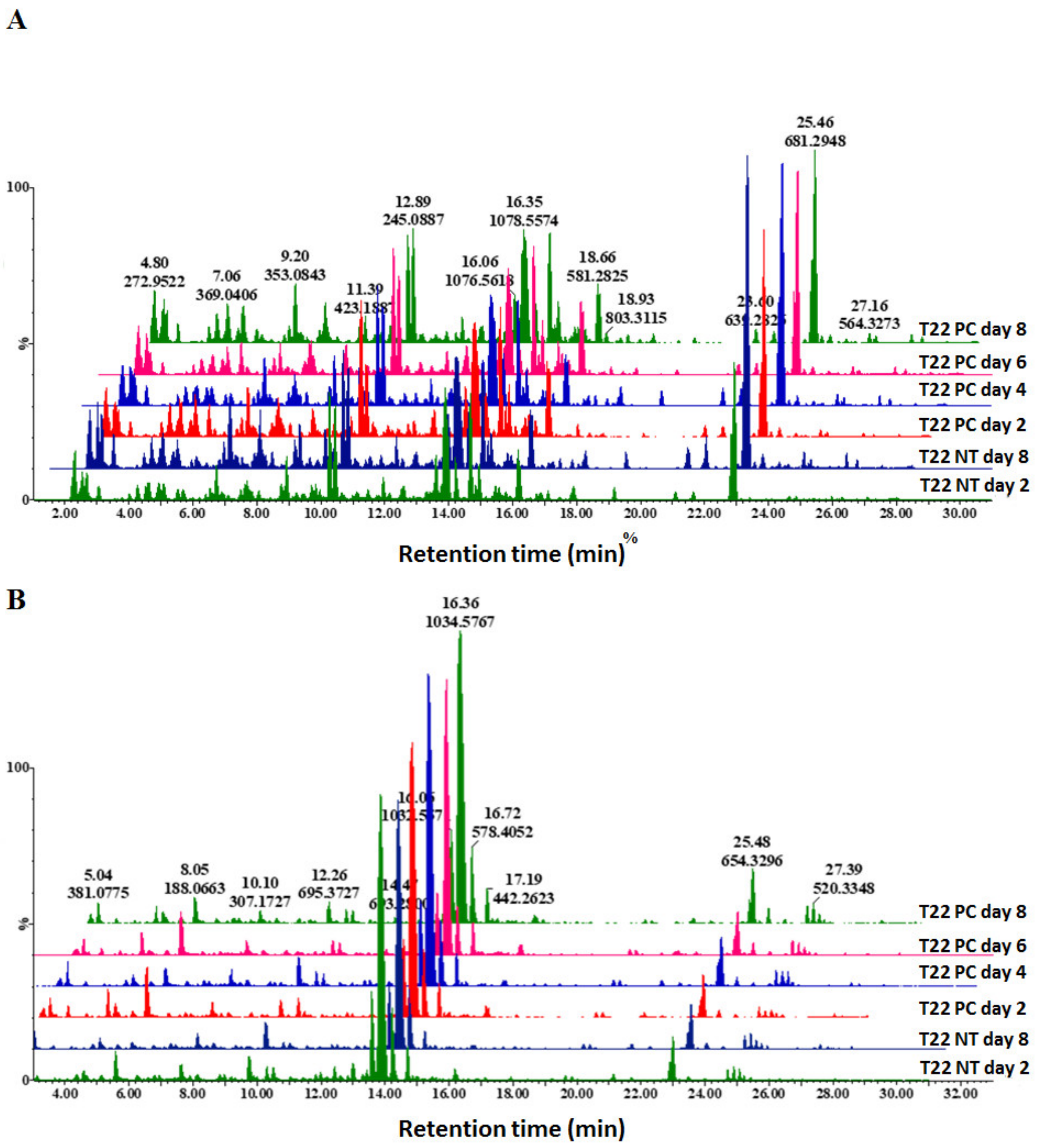

Figure S1. Representative BPI MS chromatograms of extracts from leaf tissue from primed-unchallenged (*Paenibacillus alvei* T22 NT), and primed-challenged (*Paenibacillus alvei* T22 PC) tomato plants. Base peak mass chromatograms displaying comparative chromatographic differences at different time points: primed-unchallenged (T22 NT, days 2 and 8) and primed-challenged (T22 PC, days 2, 4, 6 and 8) infected. Visual inspection of the chromatograms evidently shows differential peak populations, for instance in the **4-20 min** chromatographic region. **(A):** ESI negative mode and **(B):** ESI positive mode.

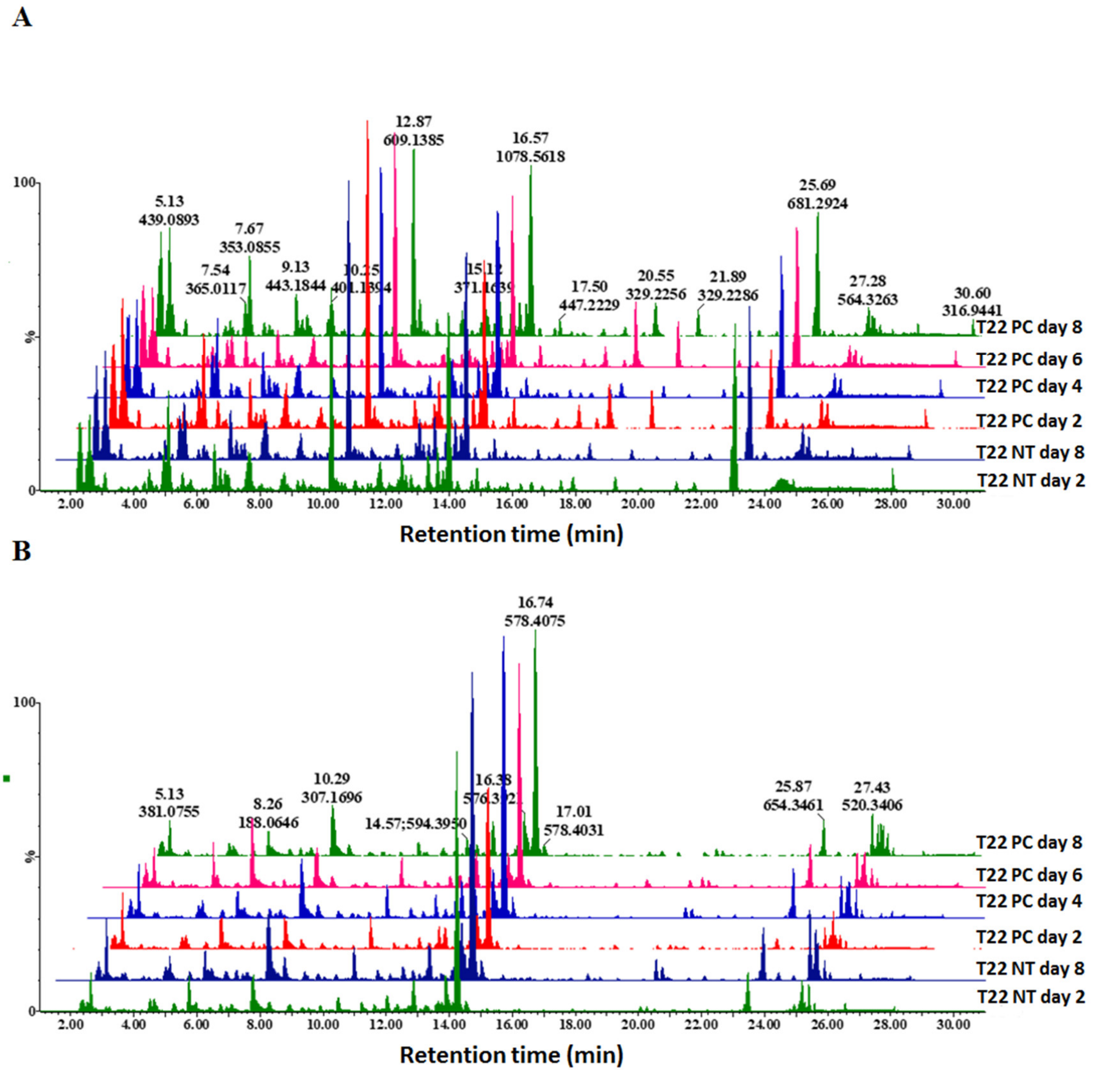

Figure S2. Representative BPI MS chromatograms of extracts from stem tissue from primed-unchallenged (*Paenibacillus alvei* T22 NT) and primed-challenged (*Paenibacillus alvei* T22 PC) tomato plants. Base peak mass chromatograms displaying comparative chromatographic differences at different time points: primed-unchallenged (T22 NT, days 2 and 8) and primed-challenged (T22 PC, days 2, 4, 6 and 8) infected. Visual inspection of the chromatograms evidently shows differential peak populations, for instance in the **4-20 min** chromatographic region. **(A):** ESI negative mode and **(B):** ESI positive mode.

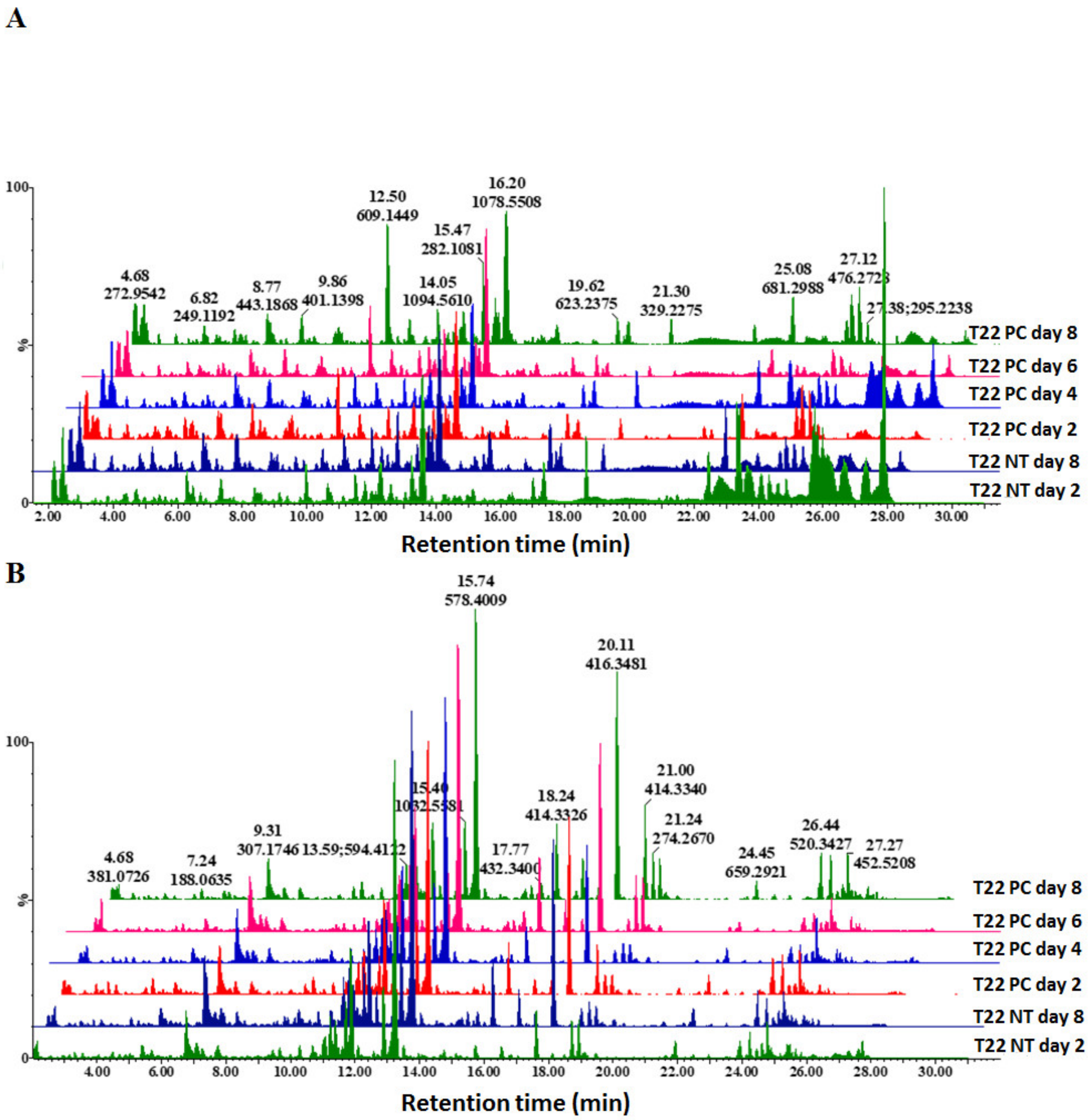

Figure S3. Representative BPI MS chromatograms of extracts from root tissue from primed-unchallenged (*Paenibacillus alvei* T22 NT) and primed-challenged (*Paenibacillus alvei* T22 PC) tomato plants. Base peak mass chromatograms displaying comparative chromatographic differences at different time points: primed-unchallenged (T22 NT, days 2 and 8) and primed-challenged (T22 PC, days 2, 4, 6 and 8) infected. Visual inspection of the chromatograms evidently shows differential peak populations, for instance in the **4-20 min** chromatographic region. **(A):** ESI negative mode and **(B):** ESI positive mode.

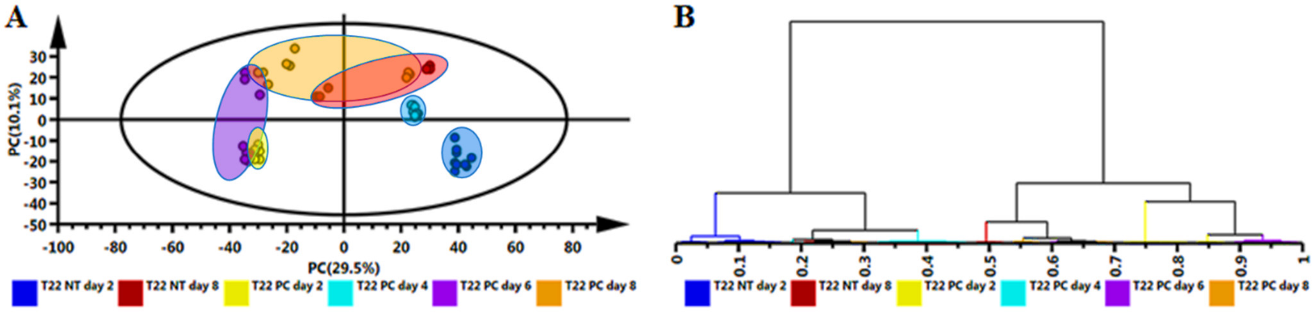

Figure S4. Unsupervised statistical analysis of tomato leaf data acquired in ESI$^+$ mode comparing *Pa. alvei* T22-primed and primed-challenged plants. (A): A PCA scores scatter plot of all the samples, including the QC samples, coloured according to time points. The PCA model presented here was a 5-component model, with R^2 of 0.54 and Q^2 of 0.41. **(B):** The HiCA dendrogram corresponding to **(A)**. Unsupervised statistical analysis is used to generate subgrouping of samples based on similar observations in **(A)** while the HCA dendrogram shows the hierarchical relationship between samples **(B)**.

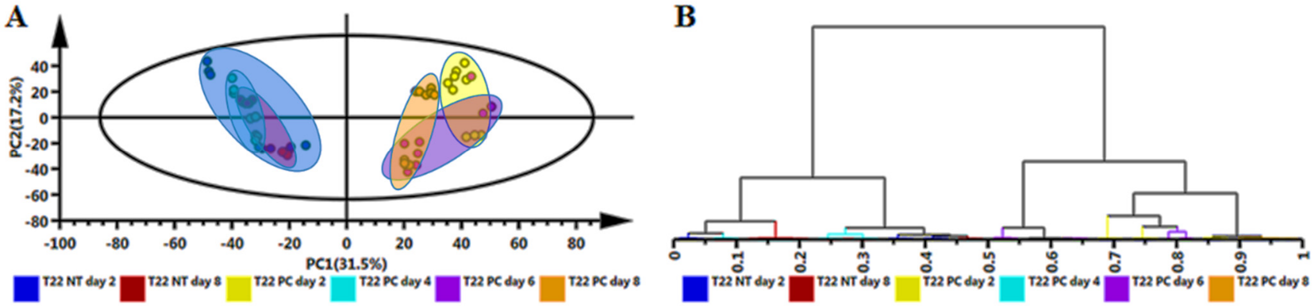

Figure S5. Unsupervised statistical analysis of tomato stem data acquired in ESI$^-$ mode comparing *Pa. alvei* T22-primed and primed-challenged plants. (A): A PCA scores scatter plot of all the samples, including the QC samples, coloured according to time points. The PCA model presented here was a 6-component model, with R^2 of 0.69 and Q^2 of 0.55. **(B):** The HCA dendrogram corresponding to **(A)**. Unsupervised statistical analysis is used to generate subgrouping of samples based on similar observations in **(A)** while the HCA dendrogram shows the hierarchical relationship between samples **(B)**.

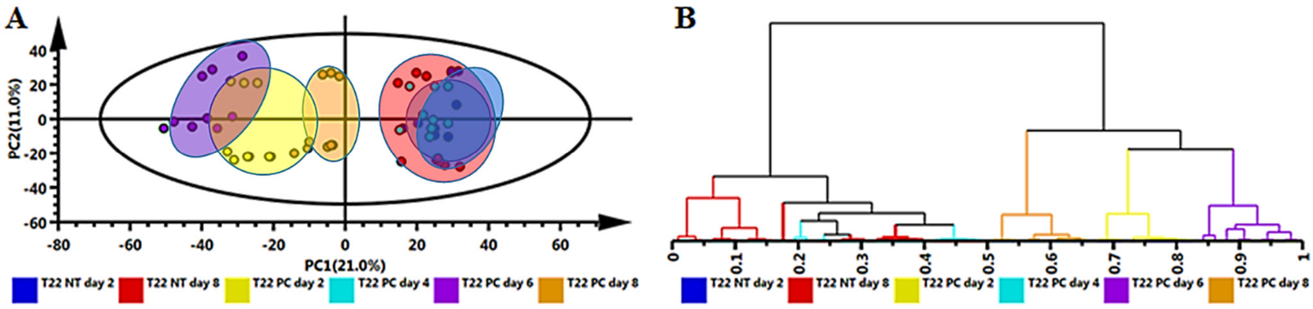

Figure S6. Unsupervised statistical analysis of tomato stem data acquired in ESI$^+$ mode comparing *Pa. alvei* T22-primed and primed-challenged plants. (A): A PCA scores scatter plot of all the samples, including the QC samples, coloured according to time points. The PCA model presented here was a 8-component model, with R^2 of 0.68 and Q^2 of 0.46. **(B):** The HCA dendrogram

corresponding to **(A)**. Unsupervised statistical analysis is used to generate subgrouping of samples based on similar observations in **(A)** while the HCA dendrogram shows the hierarchical relationship between samples **(B)**.

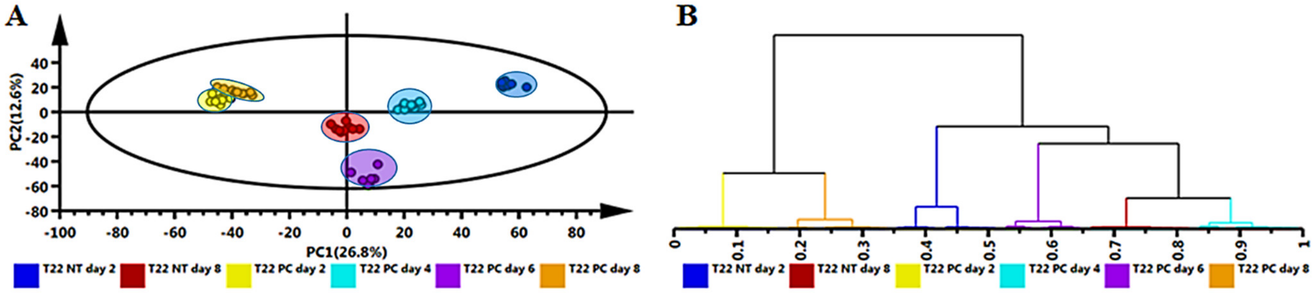

Figure S7. Unsupervised statistical analysis of tomato root data acquired in ESI^- mode comparing *Pa. alvei* T22-primed and primed-challenged plants. (A): A PCA scores scatter plot of all the samples, including the QC samples, coloured according to time points. The PCA model presented here was a 5-component model, with R^2 of 0.61 and Q^2 of 0.47. **(B):** The HCA dendrogram corresponding to **(A)**. Unsupervised statistical analysis is used to generate subgrouping of samples based on similar observations in **(A)** while the HCA dendrogram shows the hierarchical relationship between samples **(B)**.

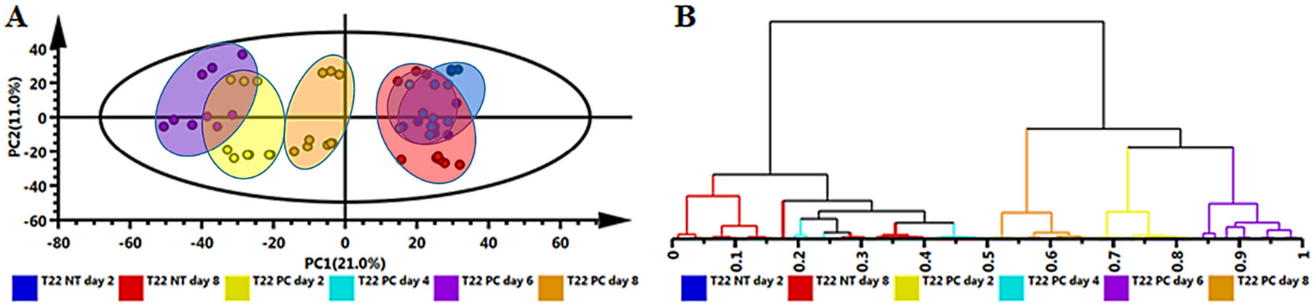

Figure S8. Unsupervised statistical analysis of tomato root data acquired in ESI^+ mode comparing *Pa. alvei* T22-primed and primed-challenged plants. (A): A PCA scores scatter plot of all the samples, including the QC samples, coloured according to time points. The PCA model presented here was a 5-component model, with R^2 of 0.78 and Q^2 of 0.61. **(B):** The HCA dendrogram corresponding to **(A)**. Unsupervised statistical analysis is used to generate subgrouping of samples based on similar observations in **(A)** while the HCA dendrogram shows the hierarchical relationship between samples **(B)**.

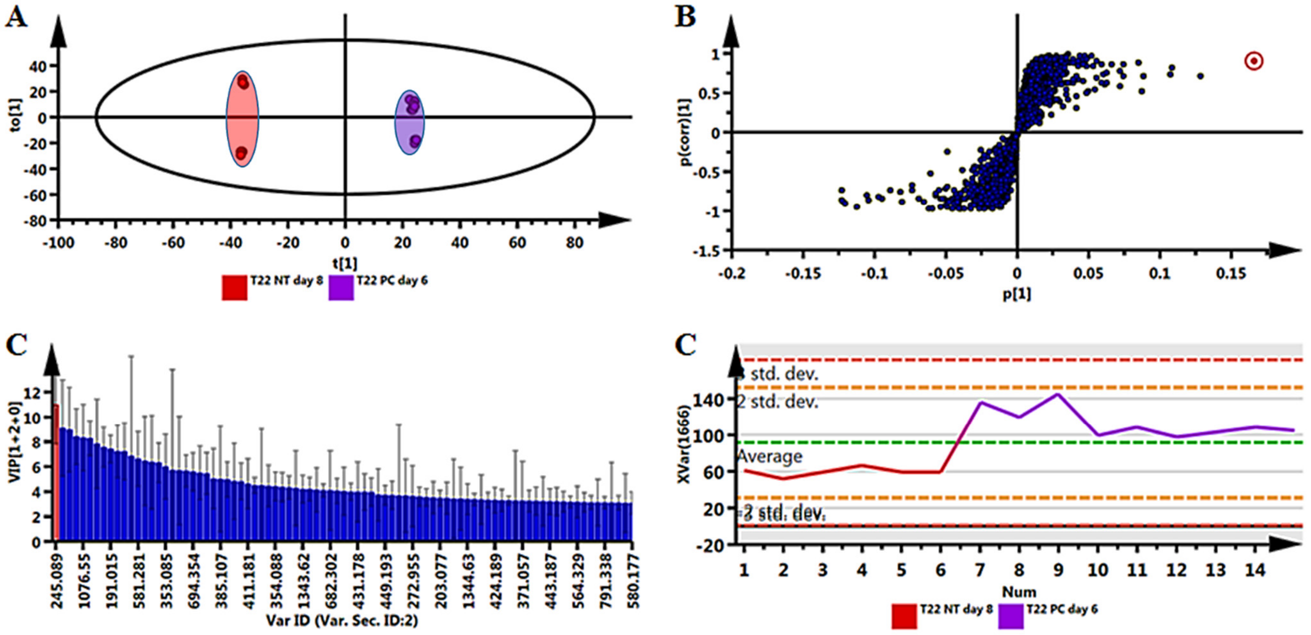

Figure S9. OPLS-DA modeling and variable/feature selection of leaf data acquired in ESI$^+$ mode comparing primed (*Paenibacillus alvei* T22 NT) and primed-challenged (*Paenibacillus alvei* T22 PC) plants. (**A**) A typical **OPLS-DA score** separating primed (T22 NT day 8) plants *vs.* primed-challenged (T22 PC day 6) plants (1 +2 + 0 components, $R^2X = 0.511$, $Q^2 = 0.946$, CV-ANOVA *p*-value = 1.4×10^{-8}). (**B**) An OPLS-DA **loadings S-plot** for the same model in (**A**); only variables with a correlation *[p(corr)]* of ≥ 0.5 and covariance of *(p1)* ≥ 0.5 were chosen as discriminating variables and identified using the *m/z* to generate an elemental composition. (**C**) **Variable importance for the projection (VIP) plot** for the same model, pointing mathematically to the importance of each variable in contributing to group separation in the OPLS-DA model. (**D**) A typical **variable trend plot** (of the selected variable in VIP and S-plots), displaying the changes of the selected variables across the samples (NT day 8 *vs.* PC day 6). This shows that the selected features significantly discriminate the primed-challenged from primed-unchallenged samples.

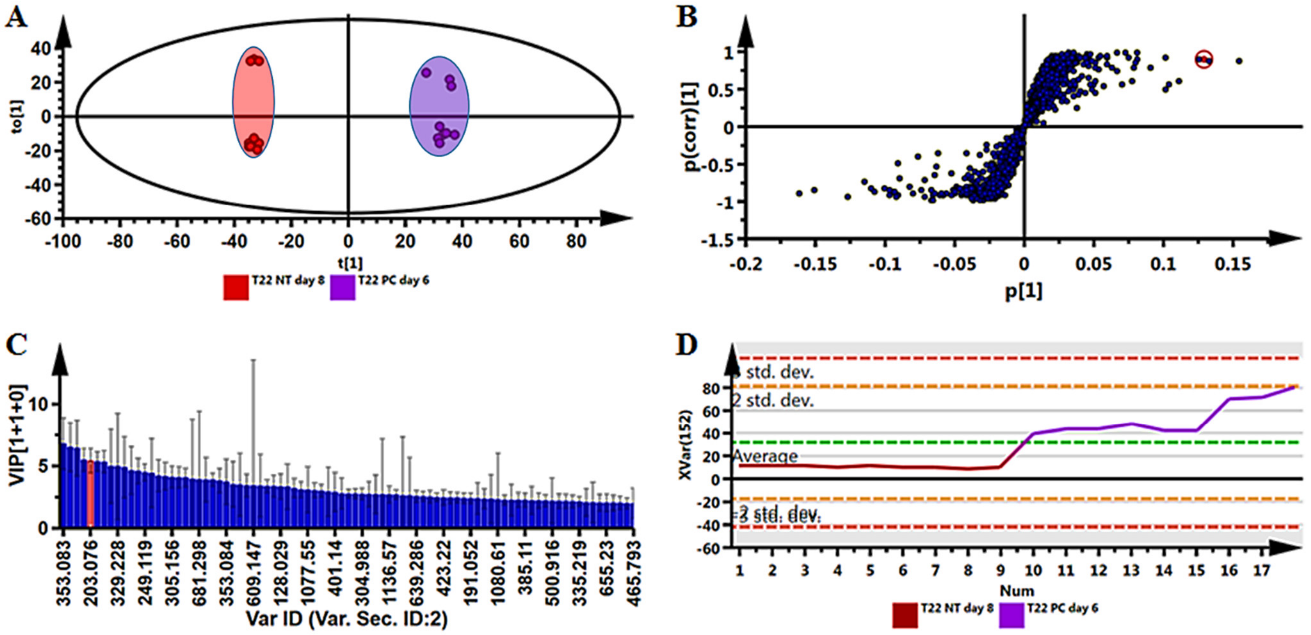

Figure S10. OPLS-DA modeling and variable/feature selection of stem data acquired in ESI^- mode comparing primed (*Paenibacillus alvei* T22 NT) and primed-challenged (*Paenibacillus alvei* T22 PC) plants. (**A**) A typical **OPLS-DA score** separating primed (T22 NT day 8) plants *vs.* primed-challenged (T22 PC day 6) plants (1 + 1 + 0 components, $R^2X = 0.462$, $Q^2 = 0.981$, CV-ANOVA *p*-value = 5.9×10^{-11}). (**B**) An OPLS-DA **loadings S-plot** for the same model in (**A**); only variables with a correlation *[p(corr)]* of ≥ 0.5 and covariance of *(p1)* ≥ 0.5 were chosen as discriminating variables and identified using the *m/z* to generate an elemental composition. (**C**) **Variable importance for the projection (VIP) plot** for the same model, pointing mathematically to the importance of each variable in contributing to group separation in the OPLS-DA model. (**D**) A typical **variable trend plot** (of the selected variable in VIP and S-plots), displaying the changes of the selected variables across the samples (NT day 8 *vs.* PC day 6). This shows that the selected features significantly discriminate the primed-challenged from primed-unchallenged samples.

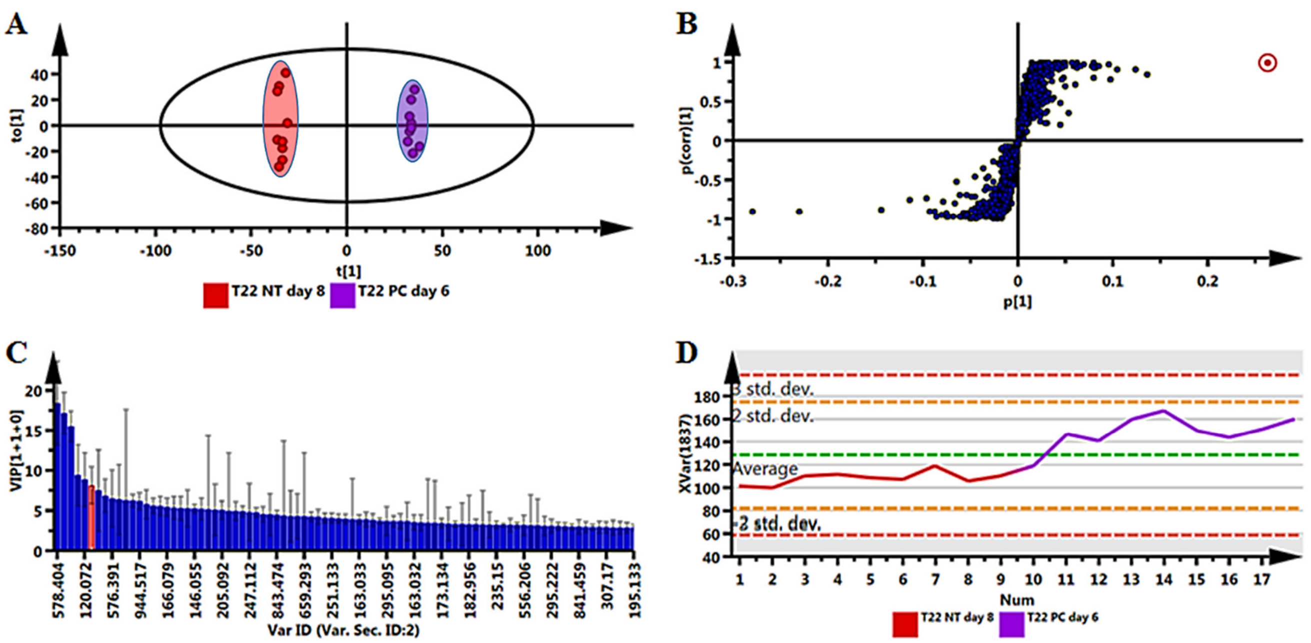

Figure S11. OPLS-DA modeling and variable/feature selection of stem data acquired in ESI$^+$ mode comparing primed (*Paenibacillus alvei* T22 NT) and primed-challenged (*Paenibacillus alvei* T22 PC) plants. (**A**) A typical **OPLS-DA score** separating primed (T22 NT day 8) plants *vs.* primed-challenged (T22 PC day 6) plants (1 + 1 + 0 components, $R^2X = 0.488$, $Q^2 = 0.984$, CV-ANOVA p-value = 1.5×10^{-11}). (**B**) An OPLS-DA **loadings S-plot** for the same model in (**A**); only variables with a correlation [*(p(corr)*] ≥ |0.6| and covariance *(p1)* ≥ |0.5| were chosen as discriminating variables and identified using the *m/z* to generate an elemental composition. (**C**) **Variable importance for the projection (VIP) plot** for the same model, pointing mathematically to the importance of each variable in contributing to group separation in the OPLS-DA model. (**D**) A typical **variable trend plot** (of the selected variable in VIP and S-plots), displaying the changes of the selected variables across the samples (NT day 8 *vs.* PC day 6). This shows that the selected features significantly discriminate the primed-challenged from primed-unchallenged samples.

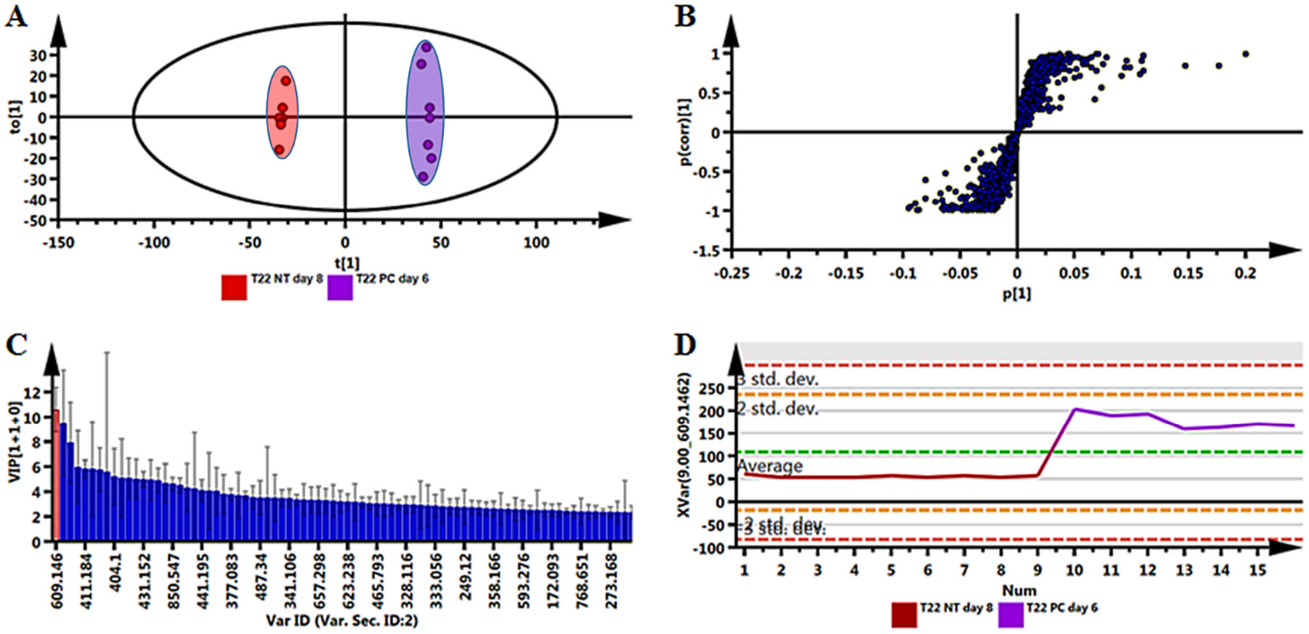

Figure S12. OPLS-DA modeling and variable/feature selection of root data acquired in ESI^- mode comparing primed (*Paenibacillus alvei* T22 NT) and primed-challenged (*Paenibacillus alvei* T22 PC) plants. (**A**) A typical **OPLS-DA score** separating primed (T22 NT day 8) plants *vs.* primed-challenged (T22 PC day 6) plants (1 + 1 + 0 components, $R^2X = 0.47$, $Q^2 = 0.976$, CV-ANOVA *p*-value = 8.1×10^{-9}). (**B**) An OPLS-DA **loadings S-plot** for the same model in (**A**); only variables with a correlation [*(p(corr)*] ≥ |0.6| and covariance *(p1)* ≥ |0.5| were chosen as discriminating variables and identified using the *m/z* to generate an elemental composition. (**C**) **Variable importance for the projection (VIP) plot** for the same model, pointing mathematically to the importance of each variable in contributing to group separation in the OPLS-DA model. (**D**) A typical **variable trend plot** (of the selected variable in VIP and S-plots), displaying the changes of the selected variables across the samples (NT day 8 *vs.* PC day 6). This shows that the selected features significantly discriminate the primed-challenged from primed-unchallenged samples.

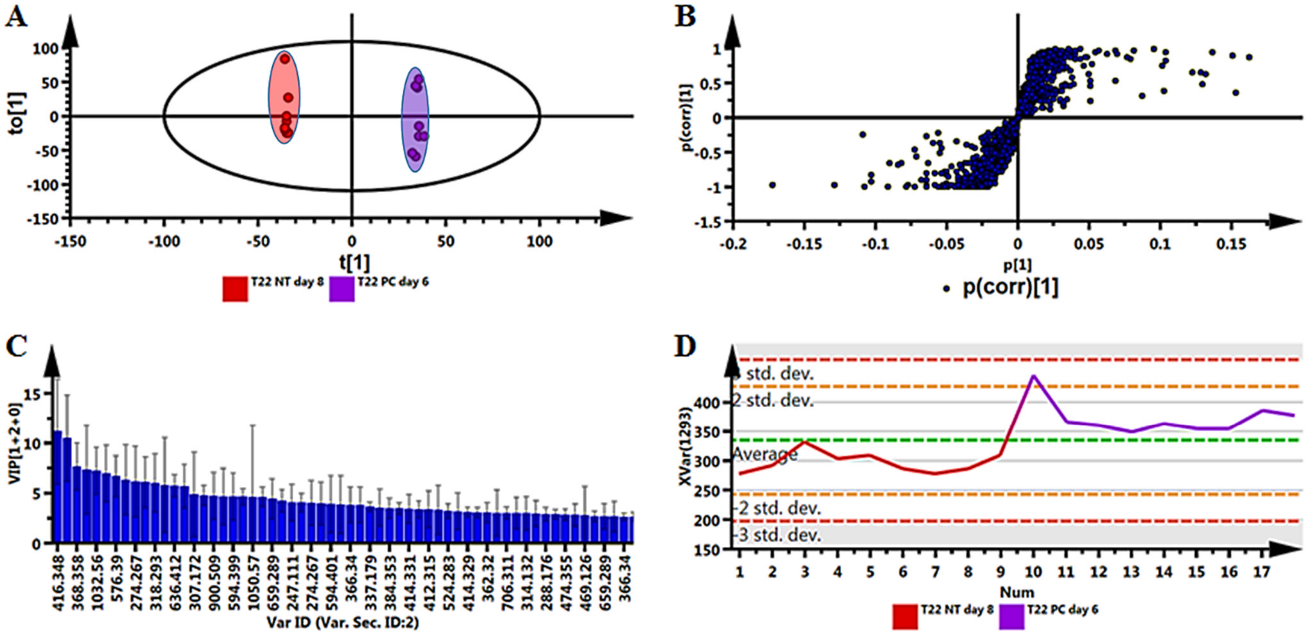

Figure S13. OPLS-DA modeling and variable/feature selection of root data acquired in ESI$^+$ mode comparing primed (*Paenibacillus alvei* T22 NT) and primed-challenged (*Paenibacillus alvei* T22 PC) plants. (**A**) A typical **OPLS-DA score** separating primed (T22 NT day 8) plants *vs.* primed-challenged (T22 PC day 6) plants (1 + 2 + 0 components, $R^2X = 0.67$, $Q^2 = 0.980$, CV-ANOVA *p*-value = 1.8×10^{-8}). (**B**) An OPLS-DA **loadings S-plot** for the same model in (**A**); only variables with a correlation [*(p(corr)*] ≥ |0.6| and covariance *(p1)* ≥ |0.5| were chosen as discriminating variables and identified using the *m/z* to generate an elemental composition. (**C**) **Variable importance for the projection (VIP) plot** for the same model, pointing mathematically to the importance of each variable in contributing to group separation in the OPLS-DA model. (**D**) A typical **variable trend plot** (of the selected variable in VIP and S-plots), displaying the changes of the selected variables across the samples (NT day 8 *vs.* PC day 6). This shows that the selected features significantly discriminate the primed-challenged from primed-unchallenged samples.
